# RNA-seq at different stages of human pancreatic β cell differentiation reveals proliferation dynamics and SMAD9 in directing β cell fate

**DOI:** 10.1038/s41419-026-08529-z

**Published:** 2026-03-10

**Authors:** Euodia Xi Hui Lim, Gabriel Jing Xiang Ong, Daniel Aron Ang, Adrian Kee Keong Teo

**Affiliations:** 1 Stem Cells and Diabetes Laboratory, Institute of Molecular and Cell Biology (IMCB), Agency for Science, Technology and Research (A*STAR), Singapore, Singapore; 2 Precision Medicine Translational Research Programme (TRP), Yong Loo Lin School of Medicine, National University of Singapore, Singapore, Singapore; 3 Department of Biochemistry, Yong Loo Lin School of Medicine, National University of Singapore, Singapore, Singapore; 4 Department of Medicine, Yong Loo Lin School of Medicine, National University of Singapore, Singapore, Singapore

**Keywords:** Differentiation, Stem-cell differentiation

## Abstract

Human pluripotent stem cells (hPSCs), such as human embryonic stem cells (hESCs) and human induced pluripotent stem cells (hiPSCs), have been successfully differentiated into pancreatic β-like cells for disease modeling and intended cell replacement therapy. These differentiating human pancreatic cells provide important insights into human pancreas development, given the difficulty in accessing human fetal pancreatic tissue. Although in-depth transcriptomic analyses, such as RNA-sequencing (RNA-Seq), have been conducted, insights into pancreatic developmental dynamics and the discovery of new pancreatic gene functions remain limited. Here, we analyzed the developmental dynamics of differentiating β-like cells and identified transcription factor signatures involved in the transition from pancreatic progenitors to endocrine progenitors, and then to β-like cells. We identified and demonstrated multiple cell cycle genes to be downregulated during late-state pancreatic β cell differentiation, accounting for their decreased proliferation during maturation. We further identified and characterized the role of yet-unreported SMAD9 in contributing toward human β cell identity and insulin secretion function. Overall, we report a rich resource of transcription factor signatures uniquely up- or downregulated during human pancreatic β cell differentiation that can be further tapped into for pancreatic biology and gene discovery.

## Introduction

Deciphering the molecular pathways guiding human pancreatic development is critical for elucidating diabetes disease mechanisms and supporting translational efforts using human pluripotent stem cells (hPSCs). The scarcity and complexity of using post-implantation human embryo samples for research have limited many developmental studies to non-human models, particularly mice [1, 2]. While it is invaluable to use mouse models to study pancreatic development, there exist differences between mouse and human pancreatic β cells. For instance, mature mouse β cells downregulate MafB and upregulate MafA, but both MAFA and MAFB are expressed in human β cells [3, 4]. Mouse β cells require a period of postnatal maturation, while human β cells have functional secretory capabilities at birth [5, 6]. In addition, mouse models do not recapitulate several human monogenic diabetes phenotypes accurately [7, 8]. Such species-specific distinctions rationalize a continued need to harness the use of human models to study human pancreas development and diabetes disease pathogenesis. In this regard, the differentiation of human embryonic stem cells (hESCs) or donor-derived human induced pluripotent stem cells (hiPSCs) into pancreatic β-like cells has shown to recapitulate in vivo β cell development, making it a suitable model to further study pathways that govern human pancreas development [9–12].

Transcriptome profile comparisons between in vitro hPSC-derived pancreatic progenitors and in vivo human embryonic pancreatic buds have demonstrated a high level of similarity, supporting the notion that in vitro differentiation recapitulates pancreatic development and is suitable to be leveraged on to reveal new regulators of pancreatic differentiation [3, 13]. While transcriptomic characterizations of hPSC-derived pancreatic β-like cells to evaluate their similarities to human islets have been made [14–17]. The transition from pancreatic progenitors to endocrine progenitors, to β-like cells, remains poorly understood. Efforts have been made to uncover the mechanisms involved in development from the mouse pancreatic bud E9.5 stage to secondary transition and beyond, but our understanding of late-stage β cell development remains primitive [18]. It is known that key signaling pathways such as the TGFβ family, WNT and Hippo signaling are involved, but the mechanisms of pathway crosstalk leading to transcription factor cascades remain elusive [13, 19–22].

We recently differentiated hESCs and hiPSCs into pancreatic β-like cells and performed bulk RNA-Sequencing (RNA-Seq) analyses at key pancreatic development timepoints—pancreatic progenitors, endocrine progenitors and β-like cells [10, 23]. While both hPSC models are highly similar, analyzing them together can reduce model- and cell line-specific effects. Here, we compared and derived a list of genes and bioprocesses that are commonly up- or downregulated in both hESC- and hiPSC-derived pancreatic cells at the various developmental timepoints. We then triaged a comprehensive list of transcription factors that are involved in this transition from pancreatic progenitors to endocrine progenitors to β-like cells, based on a reported list of human transcription factors [24]. We found multiple cell cycle genes to be downregulated during late-state pancreatic β cell differentiation, accounting for their decreased proliferation during maturation. We further identified and characterized the role of yet-unreported SMAD9 during human β cell differentiation, followed by knockdown studies in differentiating endocrine progenitors to β-like cells. Overall, our work provides a valuable and important resource for the identification of gene targets, pathways and transcriptional regulators that are uniquely up- or downregulated during human pancreatic β cell differentiation. These genes can be further explored to unravel insights into human pancreatic β cell biology.

## Results

### Identification of genes uniquely upregulated in differentiating human pancreatic endocrine progenitors and β-like cells in vitro

We previously differentiated wild-type (WT) H9 hESCs and various healthy Asian Chinese hiPSCs into pancreatic β-like cells, and performed RNA-Seq analyses [10, 23]. To harmonize the RNA-Seq datasets from both hPSC models, we combined and reanalyzed the data, filtering for the genes with *p* < 0.05, followed by fold change (FC) > 2 on both day 20 (D20) pancreatic endocrine progenitors and day 35 (D35) pancreatic β-like cells (Supplementary Fig. 1 and Supplementary Table 1).

**Fig. 1:**
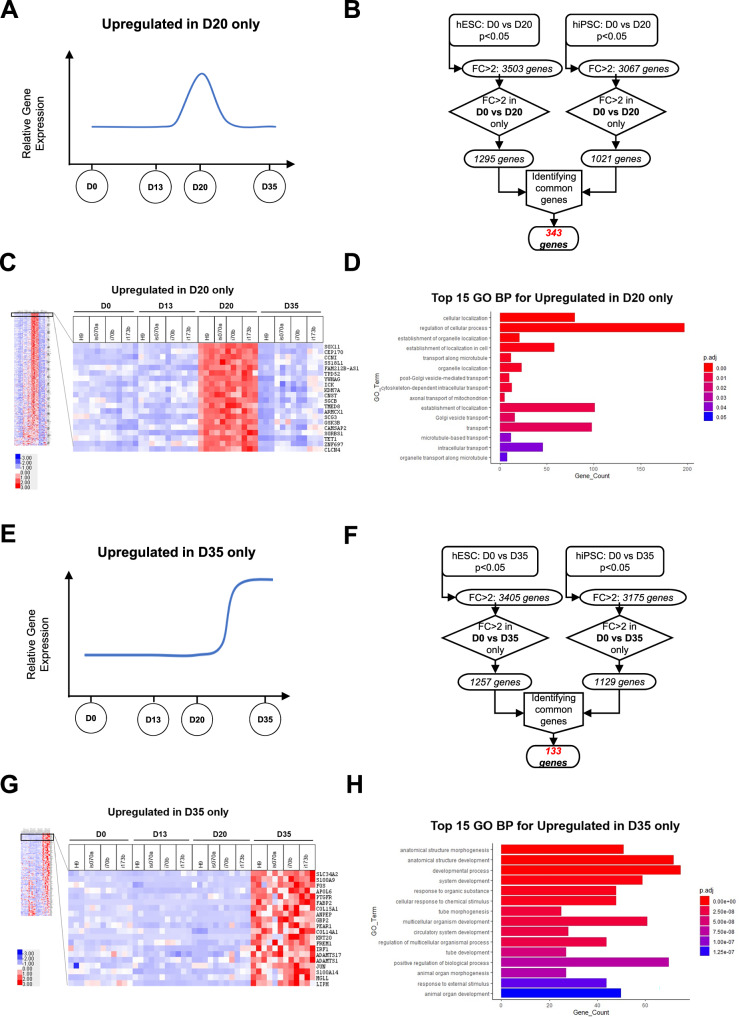
Identification of genes upregulated on D20 only and D35 only in hPSC differentiation into pancreatic β-like cells. **A** General expression profile of genes uniquely upregulated in D20 pancreatic endocrine progenitors only across the timepoints of hPSC differentiation into pancreatic β-like cells. **B** Filtering and sorting criteria for analysis of genes commonly upregulated in D20 only compared to D0 for both hESC and hiPSC differentiation into pancreatic β-like cells. **C** Heatmap of top 20 genes upregulated in D20 only in hPSC differentiation into pancreatic β-like cells. **D** Gene Ontology (GO) Biological Process (BP) analyses for genes upregulated in D20 only in hPSC differentiation into pancreatic β-like cells. **E** General expression profile of genes uniquely upregulated in D35 pancreatic β-like cells only across the timepoints of hPSC differentiation into pancreatic β-like cells. **F** Filtering and sorting criteria for analysis of genes commonly upregulated in D35 only compared to D0 for both hESC and hiPSC differentiation into pancreatic β-like cells. **G** Heatmap of top 20 genes upregulated in D35 only in hPSC differentiation into pancreatic β-like cells. **H** GO BP analyses for genes upregulated in D35 only in hPSC differentiation into pancreatic β-like cells. “See also Fig. S1”.

To better understand the differentiation process from the endocrine progenitor stage to the β-like cell stage, we first focused on genes upregulated only in D20 pancreatic endocrine progenitors (Fig. 1A) derived from both hESCs and hiPSCs, and identified 343 genes (Fig. 1B and Supplementary Table 2). We highlighted the top 20 genes that were uniquely upregulated in the endocrine progenitor stage in our heatmap analysis (Fig. 1C). Gene Ontology (GO) biological process (BP) analyses of these 343 genes indicated that these genes are generally involved in cellular localization, transport and regulation of cellular processes (Fig. 1D and Supplementary Table 2).

We repeated the same analyses to identify genes uniquely upregulated in D35 pancreatic β-like cells only, to identify genes that might be involved in the acquisition of β cell identity (Fig. 1E). We identified 133 genes that are uniquely upregulated, common in both hESC- and hiPSC-derived D35 β-like cells (Fig. 1F and Supplementary Table 2). We highlighted the top 20 genes that were uniquely upregulated in the β-like cell stage in our heatmap analysis (Fig. 1G). GO BP analyses of these 133 genes indicated that these genes are involved in response to external stimulus and multicellular organism developmental processes (Fig. 1H and Supplementary Table 2).

### Upregulation of stimulus response, regulation of hormone level genes and downregulation of neuron development genes in D20 human pancreatic endocrine progenitors differentiating toward D35 pancreatic β-like cells

**Fig. 2:**
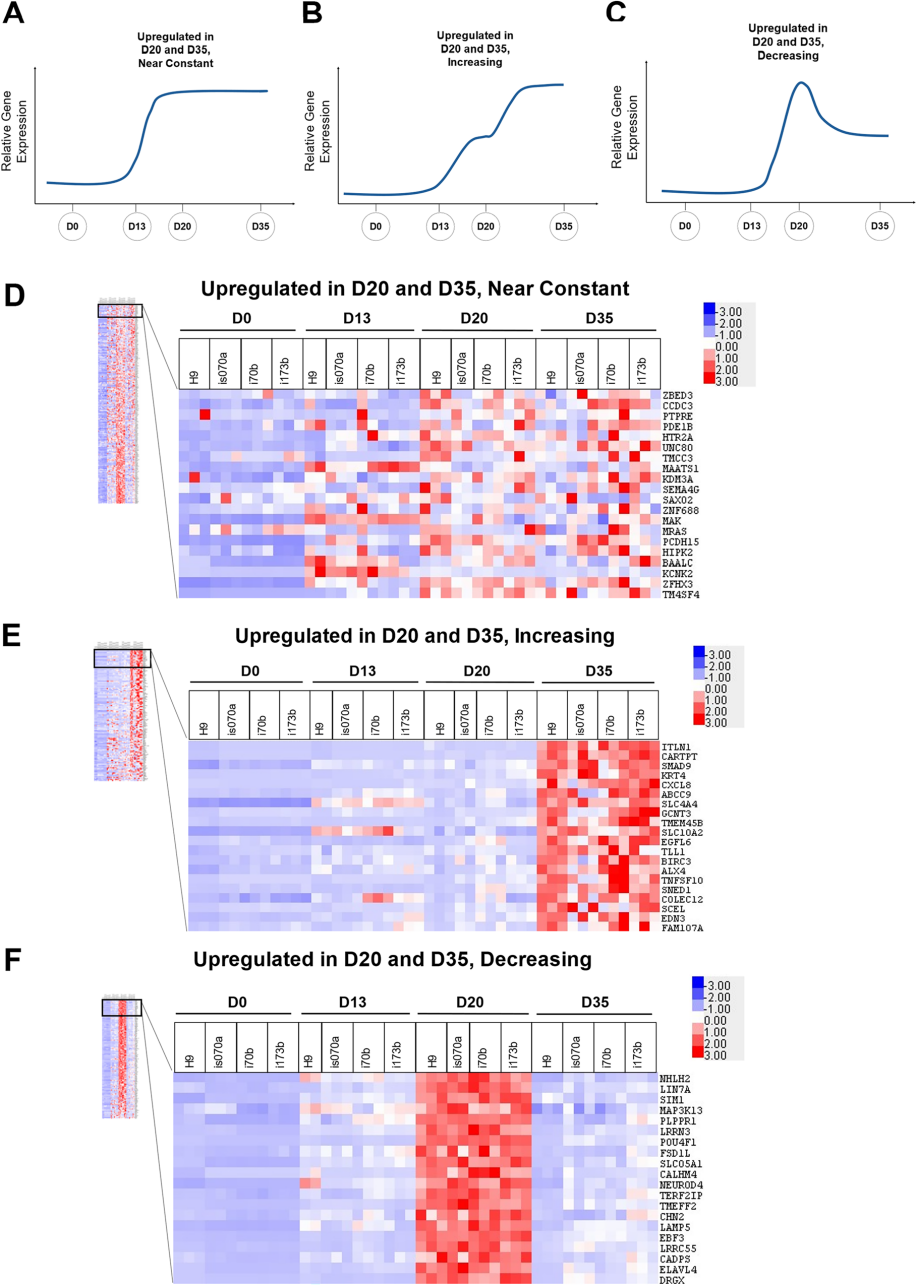
Identification of genes upregulated on D20 and D35 in hPSC differentiation into pancreatic β-like cells. General expression profile of genes upregulated in D20 and **A** near-constant, **B** increasing, and **C** decreasing at D35 across the timepoints of hPSC differentiation into pancreatic β-like cells. Heatmap of top 20 genes upregulated in D20 and **D** near-constant, **E** increasing, and **F** decreasing at D35 across the timepoints of hPSC differentiation into pancreatic β-like cells. “See also Fig. S2”.

Following the identification of genes uniquely upregulated either in D20 or D35 pancreatic cells only (Fig. 1), we sought to evaluate genes that were upregulated in both D20 and D35 pancreatic cells, which exhibited either near-constant expression, increasing or decreasing expression from D20 to D35 (Fig. 2A–C), to gain deeper insights into the biological processes and genes differentially regulated as differentiating endocrine progenitors mature to β-like cells. We identified 238 genes with near-constant increased expression from D20 to D35, 124 genes with increasing expression from D20 to D35, and 166 genes with decreasing expression from D20 to D35 (Supplementary Fig. 2A, B and Supplementary Table 3).

Heatmap analyses of the genes exhibiting near-constant increased expression were sorted. While the heatmap appeared to be “variable” (Fig. 2D), the average expression is consistently higher than undifferentiated hPSCs at D0, indicating that they are more highly expressed in D20 pancreatic endocrine progenitors and D35 pancreatic β-like cells. GO BP analyses suggested that this group of genes is generally involved in response to stimulus, signaling and developmental processes (Supplementary Fig. 2C and Supplementary Table 3).

Next, the genes exhibiting increasing expression were sorted based on the increase in average FPKM at D35 compared to D20, ordering the genes with the largest increase in expression at the top (Fig. 2E). These genes are involved in response to stimulus and regulation of hormone levels (Supplementary Fig. 2D and Supplementary Table 3), suggesting a progression from D20 pancreatic endocrine progenitors toward D35 hormone-expressing endocrine cells.

Last but not least, genes exhibiting decreasing expression, which were sorted by the decrease in average FPKM at D35 compared to D20 (Fig. 2F), are involved in nervous system development and neuron development (Supplementary Fig. 2E and Supplementary Table 3), suggesting the downregulation of neuronal-like program at D20 in favor of that of the endocrine at D35.

### D20 human pancreatic endocrine progenitors and D35 pancreatic β-like cells exhibit decreased expression of *MKI67* proliferation marker and genes involved in cell division and cell cycle

**Fig. 3:**
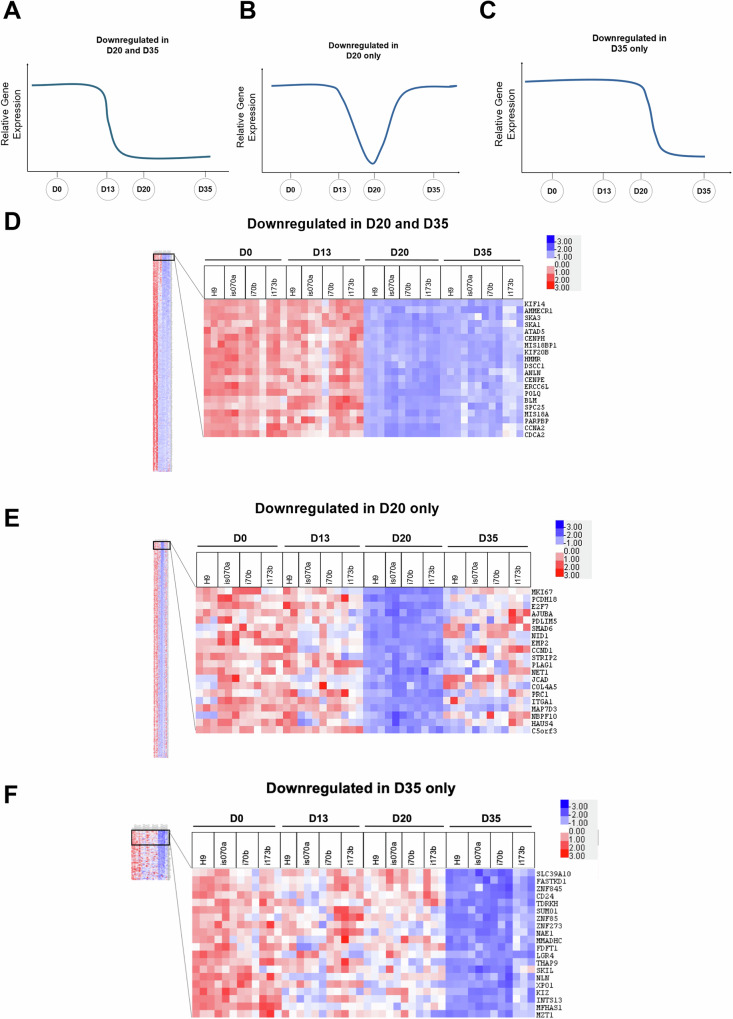
Identification of genes downregulated on D20 and D35 in hPSC differentiation into pancreatic β-like cells. General expression profile of genes downregulated in **A** D20 and D35, **B** D20 only, and **C** D35 only across the timepoints of hPSC differentiation into pancreatic β-like cells. Heatmap of top 20 genes downregulated in **D** D20 and D35, **E** D20 only and **F** D35 only across the timepoints of hPSC differentiation into pancreatic β-like cells. “See also Fig S3”.

Besides the upregulated genes that are involved in pancreatic endocrine and β cell specification, the downregulation of undesired genes is also likely to be important for pancreatic formation. We then evaluated the group of genes that were consistently downregulated in both D20 and D35 pancreatic cells (Fig. 3A), specifically in D20 (Fig. 3B) or in D35 pancreatic cells only (Fig. 3C). We identified 566 genes that were consistently downregulated in both D20 and D35, 729 genes that were only downregulated in D20, and 63 genes that were only downregulated in D35 pancreatic cells (Supplementary Fig. 3A and Supplementary Table 4).

Heatmap analyses of these three groups of downregulated genes reflected consistent expression across all the hPSC lines and their replicates (Fig. 3D–F). GO BP analyses on genes that were downregulated in both D20 and D35 interestingly indicated that they are involved in cell division and cell cycle (Supplementary Fig. 3B and Supplementary Table 4), suggesting that these D20 pancreatic endocrine progenitors and D35 β-like cells had decreased cellular proliferation from D13 onwards.

The genes that were only downregulated in D20 were broadly involved in anatomical structure development, circulatory system and metabolic process (Supplementary Fig. 3C and Supplementary Table 4). Of note, *MKI67*, which encodes for KI67 proliferation marker, was the topmost downregulated gene, indicating a downturn in cellular proliferation distinctly at D20 (Fig. 3E). There were too few genes that were only downregulated in D35. Hence, GO BP analysis was not possible.

### In vitro-derived D20 pancreatic endocrine progenitors exhibit decreased cellular proliferation reminiscent of maturing β cells

**Fig. 4:**
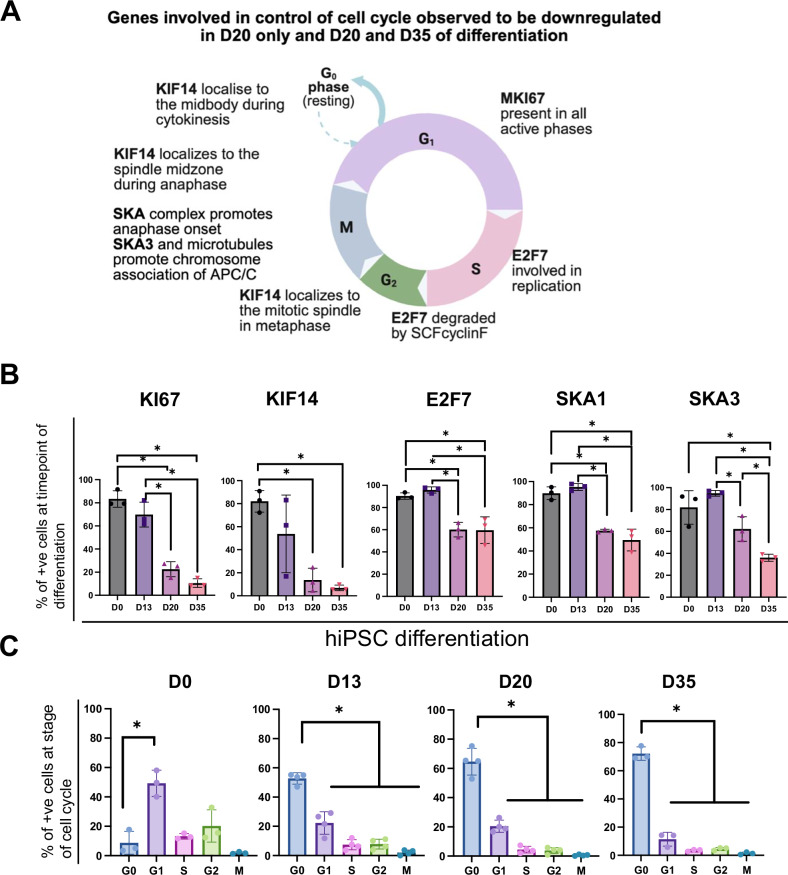
Characterization of cell cycle genes in hPSC differentiation into pancreatic β-like cells. **A** Schematic of the involvement of cell cycle genes *MKI67*, *KIF14*, *E2F7*, *SKA1* and *SKA3* in hPSC differentiation into pancreatic β-like cells. **B** Flow cytometry analyses of KI67, KIF14, E2F7, SKA1 and SKA3 across the timepoints of hiPSC differentiation into pancreatic β-like cells. Results are from *n* = 3 independent experiments. Statistical analyses were conducted with one-way ANOVA with Tukey’s post hoc test and considered to be statistically significant when *P* ≤ 0.05. **C** Cell cycle flow cytometry analyses across the timepoints of hiPSC differentiation into pancreatic β-like cells. Results are from *n* = 3 independent experiments. Statistical analyses were conducted with one-way ANOVA with Tukey’s post hoc test, and considered to be statistically significant when *P* ≤ 0.05. For all statistical analyses: Error bars represent standard deviation (STDEV).“See also Fig S4”.

Several of the top genes downregulated in D20 and D35 (Fig. 3D), and downregulated in D20 only (Fig. 3E), such as *KIF14*, *SKA3*, *SKA1, MKI67* and *E2F7* are genes involved in the cell cycle (Fig. 4A). We sought to further characterize this phenomenon of reduced proliferation as it has yet-to-be described during human β cell differentiation and maturation. qRT-PCR analyses confirmed that these genes were clearly downregulated at D20 (Supplementary Fig. 4A), with many of them continuing to be expressed at low levels compared to D0. Additional flow cytometry analyses of KI67, KIF14, E2F7, SKA1 and SKA3 further confirmed a decrease in proportion of cells positive for these proteins on D20 and D35 (Fig. 4B). Cell cycle analyses via flow cytometry then revealed that majority of the D0 cells were in the G_1_ phase of cell division, whereas D13, D20, D35 cells had exited the cell cycle and were mostly in the G_0_ stage, with the proportion of cells in G_0_ stage increasing from 50 to 70% as pancreatic differentiation progressed toward D35 mature β-like cells (Fig. 4C and Supplementary Fig. 4B, C), suggesting a switch from proliferative pluripotent stem cells to an increasing majority of quiescent cells from the pancreatic progenitor stage at D13 to D35 β-like cells.

### Transcription factors up- and downregulated during the differentiation of hPSCs into human pancreatic endocrine progenitors and β-like cells

Transcription factors are known to be important for determining cell fate specification. While several pancreatic transcription factors mediating pancreas development, such as *PDX1*, *PTF1A*, pancreatic β cells, such as *NKX6.1* and *MAFA* are well-described, the late-stage human pancreatic transcription factor network remains to be further defined [25]. We decided to leverage our D20 and D35 gene lists (Supplementary Tables 2–4) to identify new transcription factors involved in specifying human pancreatic endocrine progenitors and β-like cells.

**Fig. 5:**
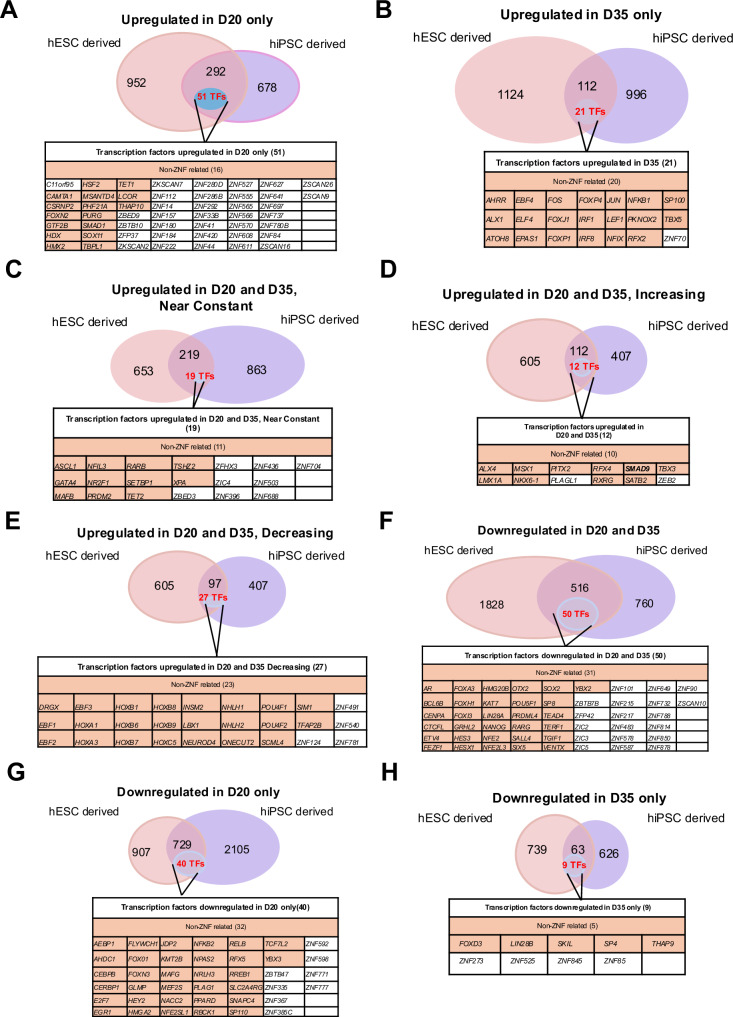
Transcription factors differentially expressed in D20 and D35 in hPSC differentiation into pancreatic β-like cells. Venn diagram of genes and list of transcription factors upregulated in **A** D20 only, **B** D35 only, D20 and **C** near constant, **D** increasing, and **E** decreasing at D35 in hESC and hiPSC differentiation into pancreatic β-like cells. Venn diagram of genes and list of transcription factors downregulated in **F** D20 and D35, **G** D20 only, and **H** D35 only in hESC and hiPSC differentiation into pancreatic β-like cells. For all statistical analyses: Error bars represent standard deviation (STDEV).“See also Fig. S5”.

We filtered the D20 and D35 up- and downregulated gene lists (Supplementary Fig. 5A and Supplementary Tables 2–4) against a reported list of transcription factors [24, 26]. and identified genes within each category that are transcription factors (Fig. 5). Amongst the eight categories explored—upregulated in D20 only, upregulated in D35 only, upregulated in D20 and D35 near constant, increasing, decreasing, downregulated in D20 and D35, downregulated in D20 only and in D35 only, the percentage of genes that make up transcription factors from all categories falls within 5–17% (Fig. 5A–H and Supplementary Fig. 5B), suggesting that transcription factors make up a small proportion of the transcriptome but play outsized regulatory roles during differentiation. We next focused on transcription factors that were differentially regulated across the various stages of differentiation, with the aim of characterizing the key biological processes observed during β cell development and identifying new regulators that may play critical roles in β cell fate specification.

There were 51 transcription factors upregulated in D20 pancreatic cells only (Fig. 5A), 21 transcription factors upregulated in D35 β-like cells only (Fig. 5B) and 12 transcription factors commonly increasingly upregulated from D20 endocrine progenitors to D35 β-like cells from hESCs and hiPSCs (Fig. 5D). Amongst these genes were *NKX6.1*, which has known roles in β cell development [27]. (Fig. 5D). There were 27 transcription factors upregulated at D20 but with decreased expression at D35 (Fig. 5E).

There were 50 transcription factors downregulated in both D20 and D35 cells (Fig. 5F). Amongst which, pluripotency factors such as *SOX2*, *SALL4*, *LIN28A*, *NANOG* and *POU5F1* were expectedly downregulated [26]. Many ZNFs of unknown biological roles were also downregulated. GO BP analyses revealed that transcription and regulation of RNA polymerase II were the most downregulated, alongside DNA-templated transcription (Supplementary Fig. 5C). There were 40 transcription factors downregulated only in D20 cells (Fig. 5G). Top 15 biological progresses in GO analysis included terms such as DNA-templated transcription, RNA biosynthetic process, nucleobase-containing compound biosynthetic processes, suggesting a decrease in cellular activity involving transcription and metabolism (Supplementary Fig. 5D). There were nine transcription factors downregulated only in D35 cells (Fig. 5H). These results agree with the GO analyses for genes downregulated in D20 and D35, and D20 only, which suggest a decrease in cellular proliferation due to a decrease in cell cycle activity. The downregulation of transcription and regulation of RNA polymerase II for transcription factors downregulated in both D20 and D35 is expected, as RNA polymerase II is known to regulate cell cycle progression [28, 29].

### SMAD9 is a newly-identified transcription factor relevant in establishing human pancreatic β cell identity

**Fig. 6:**
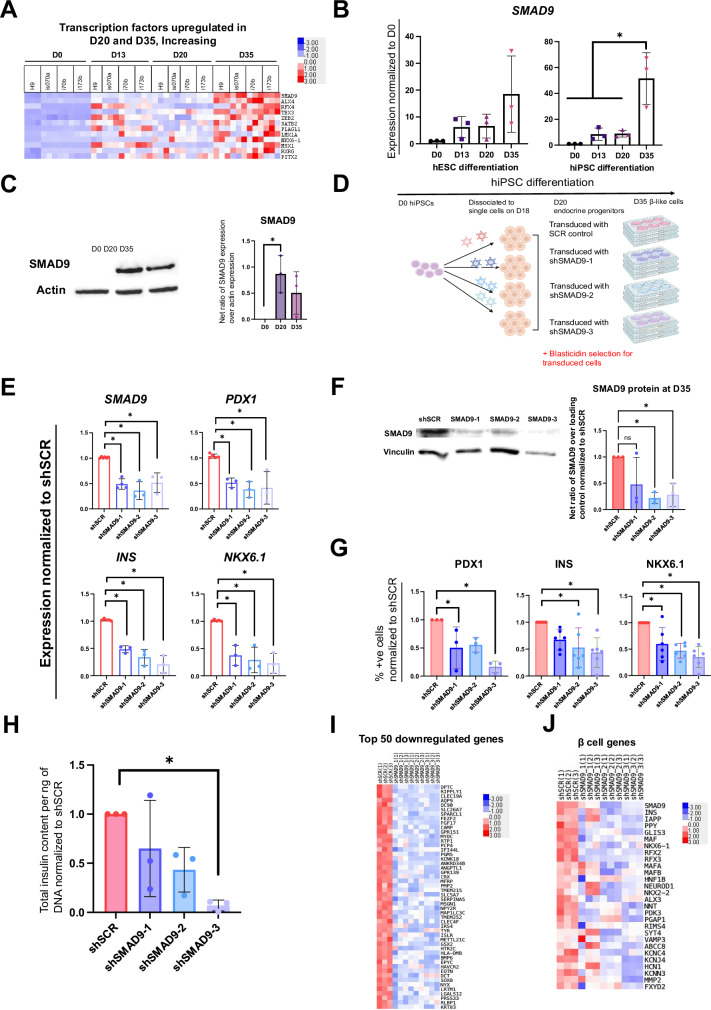
Expression and function of SMAD9 during hPSC differentiation into pancreatic β-like cells. **A** Heatmap of transcription factors upregulated in D20 and increasing at D35 across the timepoints of hPSC differentiation into pancreatic β-like cells. **B** qRT-PCR analyses of *SMAD9* during hESC and hiPSC differentiation into pancreatic β-like cells. Results are from *n* = 3 independent experiments. Statistical analysis conducted with one-way ANOVA with Tukey’s post hoc test and considered to be statistically significant when *P* ≤ 0.05. **C** Western blot analyses of SMAD9 protein levels during hiPSC differentiation into pancreatic β-like cells. Results are from *n* = 3 independent experiments. Statistical analysis conducted with one-way ANOVA with Turkey’s post hoc test and considered to be statistically significant when *P* ≤ 0.05. **D** Schematic of experimental setup to study the effect of knocking down *SMAD9* from D20 during hiPSC differentiation into pancreatic β-like cells. **E** qRT-PCR analyses of *SMAD9* and key β cell markers *PDX1*, *INS,* and *NKX6.1*. **F** Western blot analyses of SMAD9 protein levels. **G** Flow cytometry analyses of PDX1, INS and NKX6.1, and **H** total insulin content in D35 β-like cells, where *SMAD9* is knocked down from D20. Results are from *n* = 3 independent experiments. Statistical analysis for **E**–**H** was conducted with one-way ANOVA with Dunnett’s post hoc test and considered to be statistically significant when *P* ≤ 0.05. Heatmap of **I** top 50 genes and **J** β cell genes downregulated in D35 β-like cells, where *SMAD9* is knocked down from D20. For all statistical analyses: Error bars represent standard deviation (STDEV).“See also Fig. S6”.

From our transcription factor analyses (Fig. 5), *SMAD9* emerged as the top amongst 12 transcription factors (including *TBX3*, *NKX6.1*) to be increasingly expressed from D20 to D35 cells (Figs. 5D, 6A and Supplementary Fig. 6A). qRT-PCR analyses confirmed *SMAD9* transcripts to increase on D13 and D20, with peak expression in D35 β-like cells in both hESCs and hiPSCs (Fig. 6B). Western blot analyses then confirmed that it is highly expressed in both D20 and D35 pancreatic cells compared to D0 (Fig. 6C).

Given the newly-identified high expression of SMAD9 in D20 endocrine progenitors and D35 β-like cells, we wondered if SMAD9 transcription factor might play a role in the specification of human endocrine progenitors to β-like cells. Hence, we used shRNAs to knockdown *SMAD9* in differentiating D20 human pancreatic endocrine progenitors to determine its effects on subsequent development into D35 β-like cells (Fig. 6D). Successful knockdown of *SMAD9* via three independent shRNAs (Supplementary Fig. 6B) interestingly resulted in a follow-on significant decrease in key pancreatic genes *PDX1*, *INS* and *NKX6.1* when analyzed by qRT-PCR on D35 cells (Fig. 6E). This reduction in SMAD9 protein was validated via western blot (Fig. 6F) and, reduction in PDX1, INS and NKX6.1 protein were validated via flow cytometry analyses (Fig. 6G and Supplementary Fig. 6C). SMAD9 knocked down cells also had significantly lower total insulin content per ng of DNA when compared to shSCR control D35 β-like cells (Fig. 6H).

To then determine the genome-wide effects of SMAD9 knockdown, we performed RNA-Seq analyses on these D35 β-like cells. Principal component analyses (PCA) confirmed a clear segregation between shSCR and shSMAD9 D35 β-like cells (Supplementary Fig. 6D). After filtering for genes with *P* adjusted <0.05 and absolute FC > 0.5, a total of 1892 genes were commonly downregulated, and 1535 genes were commonly upregulated across all three shSMAD9 knocked down D35 β-like cells (Supplementary Table 5). *SMAD9* was reassuringly downregulated in these cells (Supplementary Fig. 6E). We next focused on the common genes downregulated in SMAD9-knockdown D35 β-like cells (Fig. 6I, J) to evaluate the consequences of the reduction of SMAD9 on β cell differentiation. GO BP analyses on the downregulated genes revealed that SMAD9 knockdown generally affected multicellular organismal and developmental processes (Supplementary Fig. 6F).

Further scrutiny on β cell genes [16]. showed that the knockdown of SMAD9 resulted in a decrease in the expression of many genes known to be important for β cell identity or function, including *INS*, *IAPP*, *GLIS3*, *NKX6.1*, *RFX2*, *RFX3*, *MAFA*, *MAFB*, *HNF1B*, *NEUROD1*, *NKX2.2*, ion channel and membrane-related genes *VAMP3*, *ABCC8*, *KCNC4*, *KCNJ4*, *KCNN3*, *FXYD2* that are known to be important for insulin secretion (Fig. 6J). These results collectively reflect the importance of SMAD9 transcription factor in the acquisition of β cell fate from human pancreatic endocrine progenitors.

We also analyzed the transcript profiles of the other SMADs to determine if *SMAD9* is unique to D35 β cells. *SMAD9* was found to be the only *SMAD* to be increasingly expressed at D35 of β cell differentiation (Supplementary Fig. 6G), with the other *SMAD*s and their BMP ligands (Supplementary Fig. 6H) that are known to interact with BR-SMADs having variable expression patterns throughout pancreatic differentiation. The other R-SMADs, *SMAD1* and *SMAD5*, did not exhibit the same expression profile as *SMAD9* during human β cell differentiation (Supplementary Fig. 6G), suggesting a possible unique role for SMAD9 in comparison to other members of the same SMAD family, in the acquisition of β cell identity during maturation from endocrine progenitors to β-like cells.

### SMAD9 is functionally relevant in EndoC-βH1 human β cells

**Fig. 7:**
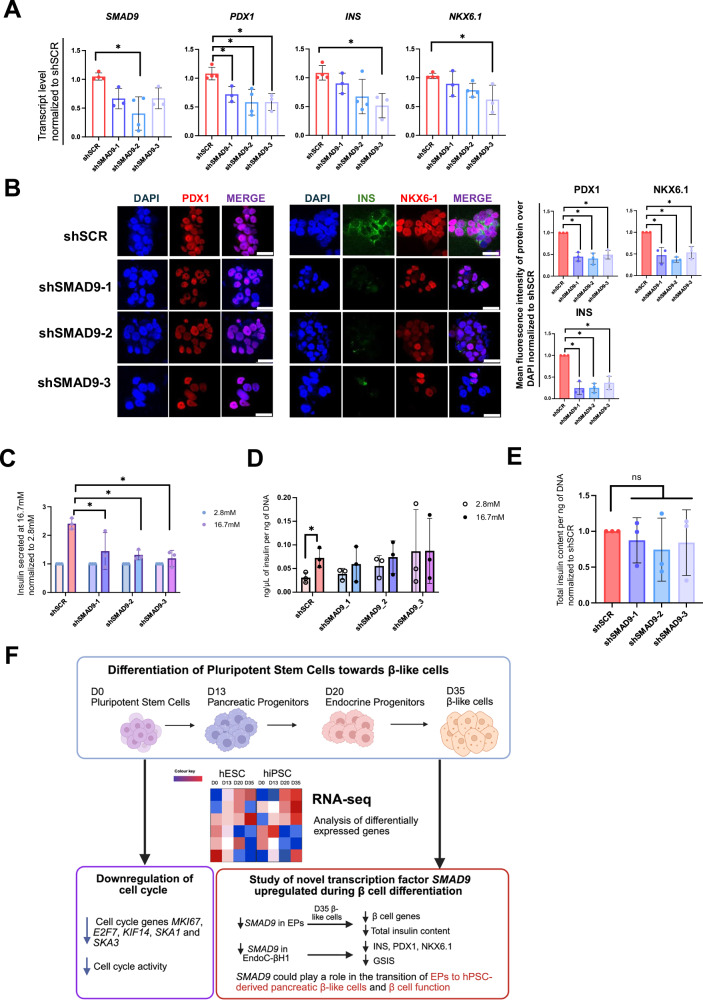
Knockdown of *SMAD9* in EndoC-βH1 cells. **A** qRT-PCR analyses of *SMAD9* and key β cell markers *PDX1*, *INS,* and *NKX6.1*. **B** Immunostaining for PDX1, INS, NKX6.1, **C** GSIS assay normalized to insulin secreted at 2.8 mM, **D** GSIS assay normalized to DNA content of cells, and **E** total insulin content in EndoC-βH1 cells transiently knocked down for *SMAD9*. Results are from *n* = 3 independent experiments. Statistical analyses for **A**–**E** were conducted with one-way ANOVA with Dunnett’s post hoc test and considered to be statistically significant when *P* ≤ 0.05. Mean fluorescent intensity (MFI) of protein over MFI of DAPI is normalized to that of shSCR cells. Data is presented from the average of three readings per experiment, from three independent experiments. Scale bars: 50 µm. Statistical analyses were conducted with two-way ANOVA with Sidak’s post hoc test for **C**, **D**; and considered to be statistically significant when *P* ≤ 0.05. Results are from *n* = 3 independent experiments. **F** Summary of findings from this manuscript. For all statistical analyses: Error bars represent standard deviation (STDEV).“See also Fig. S7”.

Given that sustained SMAD9 knockdown during pancreatic differentiation impaired β cell marker expression and insulin production in hiPSC-derived D35 β-like cells, we next asked whether SMAD9 is also important in mature human β cells compared to restricted function in differentiating human endocrine progenitors. Using the same three shRNA constructs targeting *SMAD9* (Supplementary Fig. 6B), we knocked down *SMAD9* in EndoC-βH1 human β cells. qRT-PCR analyses three days post transduction showed that the knockdown of *SMAD9* also appeared to reduce *PDX1*, *INS*, and *NKX6.1* transcript levels (Fig. 7A). This decrease was further confirmed at the protein level via immunofluorescence staining (Fig. 7B). Glucose-stimulated insulin secretion (GSIS) functional assays then revealed that the knockdown of *SMAD9* reduced the ability of these cells to respond to high glucose, without any significant increase in insulin secretion (Fig. 7C). When normalized to DNA content, these cells were non-functional (Fig. 7D) albeit without differences in total insulin content (Fig. 7E).

### *SMAD9* is positively correlated with increased insulin secretion from human islets and is more enriched in human β than α cells

As our results suggested that SMAD9 might play a role in establishing human β cell identity, we decided to evaluate the expression profile of *SMAD9* in existing databases relevant to β cells and diabetes. We searched for *SMAD9* expression on humanislets.com, a resource of metadata derived from molecular omics and assays from 547 human islet donors [30]. Based on bulk RNA-Seq analyses, we identified *SMAD9* to be significantly associated with increasing insulin secretion from human islets at 16.7 mM glucose (Supplementary Fig. 7A). *SMAD9* transcript levels were also positively associated with an increasing proportion of β cells (Supplementary Fig. 7B) but negatively associated with an increasing proportion of α cells (Supplementary Fig. 7C). This suggests that *SMAD9* may be a human β cell enriched transcription factor. Single-cell transcriptomics analyses performed by Balboa et al. on hPSC-derived islet-like cells and human islets then demonstrated *SMAD9* to be broadly clustered in early stem cell (sc)-β cells, late sc-β cells and in adult β cells [15]. (Supplementary Fig. 7D). We then sought to reanalyze the datasets from Balboa et al., focusing on endocrine progenitor, early and late sc-β cells within the defined endocrine cluster. It was observed that the endocrine progenitor cluster is more distinctly segregated from the early and late sc-β cell clusters, while the early and late sc-β cell clusters are more overlapping and heterogenous (Supplementary Fig. 7E). Importantly, the trajectory analysis over pseudotime also suggested that *SMAD9* expression starts to peak in early sc-β cells, then declines further during maturation (Supplementary Fig. 7F).

## Methods

### RNA-Seq Preprocessing, Alignment, and Quantification

Raw paired-end RNA-Seq reads in FASTQ format were first subjected to adapter and quality trimming using Trim Galore (v0.6.6). For each sample, both forward and reverse reads were trimmed in paired-end mode, and only high-quality reads passing trimming were retained for downstream analysis.

Trimmed reads were then aligned to the human reference genome (GRCh38, GENCODE annotation; Cell Ranger GRCh38-2020-A gene set) using STAR (v2.7.2). Alignment parameters allowed retention of multi-mapping reads (--outSAMmultNmax -1), preservation of unmapped reads in paired format (--outSAMunmapped Within KeepPairs), and ordered paired-end SAM output (--outSAMorder PairedKeepInputOrder). Alignments were generated in SAM format and subsequently converted into compressed BAM files using samtools.

To quantify gene-level expression, aligned BAM files were processed with featureCounts (Subread package, v2.0.1). Gene counts were generated in paired-end mode (-p) with strand-specific counting (-s 2) restricted to exonic regions (-t exon) and grouped by gene identifiers (-g gene_id). The same GENCODE GRCh38 annotation file used for STAR alignment was applied for read summarization.

### RNA-Seq analyses and functional annotations

RNA-Seq analysis was performed using previously published data consisting of total RNA extracted from hESC- and hiPSC-differentiated β-like cells on D0 hPSCs, D20 endocrine progenitors and D35 β-like cells [10, 23]. Comparisons between reads on D0 versus D20, as well as D0 versus D35, were performed in individual hPSC datasets to obtain the relative fold change (FC) in transcripts against D0. We defined upregulation as genes with FC > 2.0, and downregulation as genes with FC < 0.5. We then stratified the genes into different categories based on whether they were differentially regulated uniquely on D20 or D35, or on both D20 and D35 compared to D0.

For genes that were upregulated on both D20 and D35, we further categorized them into three groups based on the change in FC from D20 to D35. This was calculated by dividing the FC on D35 by the FC on D20. A change in FC > 1.5, FC < 0.5, and 0.5 ≤ FC ≤ 1.5 was used to categorize genes with increasing, decreasing or near constant expression from D20 to D35 respectively. For e.g., if Gene A had a FC of 2 for D20 vs D0, and a FC of 6 for D35 vs D0, this meant that the change in FC = 6/2 = 3, and it will be grouped as a gene with an increasing expression from D0 to D20 to D35. In total, the genes were split into eight distinct categories (upregulation in D20/D35 only, upregulation in D20 and D35 (increasing/decreasing/near constant), downregulation in D20/D35 only, downregulation in D20 and D35), and common genes in each category, found in both hESC and hiPSC datasets, were identified.

Heatmaps (upregulation in D20/D35 only, downregulation in D20/D35 only, downregulation in D20 and D35) were sorted using the average FPKM values across the hESC and hiPSC lines. The other heatmaps (upregulation in D20 and D35 (increasing/decreasing/near constant)) were sorted by the difference between the average FPKM values on D20 and D35.

Transcript functional annotation was carried out with g:Profiler [31]. using Gene Ontology (GO) Biological Process (BP). We sorted terms by p-value and showed the most significant terms for each category. Results were presented using either R or Python.

### Identification of transcription factors

We identified the human transcription factors in each category by comparing each list against the compiled list of human transcription factors from Lambert, Jolma [24]. Heatmaps and functional annotations were then performed in a similar manner as previously described.

### Trajectory analysis of scRNA-Seq data from Balboa et al., 2022

The processed data from Balboa et al., 2022 was re-analyzed, focusing on the cell types of interest (endocrine progenitor, early and late sc-β cells) within the defined endocrine cluster [15]. Slingshot [32] analysis was employed for trajectory analysis of *SMAD9* from endocrine progenitor development towards early then late sc-β cells. For the analysis of *SMAD9* across endocrine progenitor to early and late sc-β cells, fold change of *SMAD9* expression in the cell type compared to the mean β cell cluster expression was obtained from [15].

### Cell culture

All cells were routinely tested to be mycoplasma negative. i70b hiPSCs previously generated and characterized by Lau et al. [10]. were used in this study. EndoC-βH1 cells were purchased from Univercell Biosolutions.

#### hPSC culture

hPSCs were cultured in TeSR™-E8™ (Stemcell™ Technologies, 05990). For regular maintenance, hPSCs were passaged either by colony picking or with ReLeSR (Stemcell™ Technologies, 100-0483) and seeded onto tissue culture-treated plates coated with 0.1% gelatin for ten min and coating media for a minimum of two days. Coating media consisted of high glucose Dulbecco’s modified Eagle’s medium (DMEM) (Hyclone, SH30243.01) supplemented with 10% heat-inactivated fetal bovine serum (FBS) (Hyclone, 11521851) and 1% Non-Essential Amino Acids (NEAA) (Invitrogen,11140050). Coating media was aspirated, and plates rinsed with 1x Phosphate Buffered Saline (PBS) prior to seeding. Culture media was changed daily.

#### Differentiation of hPSCs to D35 β-like cells and cell dissociation

One day prior to differentiation, hPSCs were washed with 1x PBS and incubated with 3 ml of TrypLE (Gibco, 12605010) for 3 min in the incubator. TrypLE was then aspirated, and the plate washed with 3 ml of mTeSR™1 (Stemcell™ Technologies, 85850). Cells were then passed through a 40 µm cell strainer to remove cell clumps. Cells were diluted to a density of one million cells/ml in mTeSR™1 supplemented with 10 μM Y-27632, and seeded at a density of 3 million cells/well in non-treated 6-well plates. The cells were then placed on a shaker at 80 rpm, and cultured at 37 °C with 5% CO_2_. The cells were allowed to aggregate to form uniform spheroids for one day before the initiation of differentiation. From D0 onwards, media was changed accordingly (with the addition of growth factors) as below by tilting the plate to allow cell clumps to sink before media was aspirated. Basal media composition is as follows – S1 media: MCDB131 (Gibco™, 10372019), 8 mM glucose (Sigma Aldrich, G8769), 2.46 g/l NaHCO_3_ (Sigma, S5761-500G), 2% FAF-BSA (Proliant, 68700), 1:50000 ITS-X (ThermoFisher Scientific, 51500-056), 2 mM GlutaMAX™ Supplement (Gibco™, 11574466), 0.25 mM Ascorbic Acid, 1% Penicillin/Streptomycin (Gibco™, 15140122). S2 media: MCDB131, 8 mM glucose, 1.23 g/l NaHCO_3_, 2% FAF-BSA, 1:50000 ITS-X, 2 mM GlutaMAX™ Supplement, 0.25 mM Ascorbic Acid (Sigma, Aldrich, A92902), 1% Penicillin/Streptomycin. S3 media: MCDB131, 8 mM glucose, 1.23 g/l NaHCO_3_, 2% FAF-BSA, 1:200 ITS-X, 2 mM GlutaMAX™ Supplement, 0.25 mM Ascorbic Acid, 1% Penicillin/Streptomycin. S5 media: MCDB131, 20 mM glucose, 1.754 g/l NaHCO_3_, 2% FAF-BSA, 1:200 ITS-X, 2 mM GlutaMAX™ Supplement, 0.25 mM Ascorbic Acid, 10 μg/ml Heparin, 1% Penicillin/Streptomycin. CMRL: CMRL 1066 Supplemented (Mediatech Inc, 99-603-CV), 1% Penicillin/Streptomycin.

#### Initiation of pancreatic differentiation

Media was changed according to the following schedule: human Pluripotent Stem Cell stage – D0: S1 supplemented with 100 ng/ml Activin (R&D systems, 338-AC-50) and 3 μM CHIR (Tocris, 4423), D1 – S1 supplemented with 100 ng/ml Activin. Definitive Endoderm stage – D3 and D5: S2 supplemented with 50 ng/ml FGF7 (MACS, 130-097-178). Primitive Gut Tube stage – D6: S3 supplemented with 50 ng/ml FGF7, 2 μM RA (Sigma, D2650-10), 0.25 μM Sant1 (Santa Cruz, sc-203253), 500 nM PDBu (Tocris, 4153), 200 nM LDN (Sigma, SML0559-5MG), D7: S3 supplemented with 50 ng/ml FGF7, 2 μM RA, 0.25 μM Sant1, 500 nM PDBu. Pancreatic Progenitor 1 stage – D8, D10, D12 – S3 supplemented with 50 ng/ml FGF7, 100 nM RA, 0.25 μM Sant1. Pancreatic Progenitor 2 stage – D13, D15: S5 supplemented with 100 nM RA, 0.25 μM Sant1, 1 μM XXI, 10 μM Alk5iII (Enzo Life Sciences, ALX-270-445), 1 μM T3 (Millipore, 642511), 20 ng/ml Betacellulin (Cell Signaling, 5235SF). D17, D19 – S5 supplemented with 25 nM RA, 1 μM XXI, 10 μM Alk5iII, 1 μM T3, 20 ng/ml Betacellulin. Endocrine Progenitor stage – D20, D22, D24, D26 and Stem Cell (SC)-derived β cell stage – D28, D30, D32, D34: CMRL supplemented with 10 μM Alk5iII and 1 μM T3.

For differentiations involving reaggregation to spheroids, media modifications from the following stages are as follows: Primitive Gut Tube stage – D6 and D7: S3v3 supplemented with 50 ng/ml FGF7, 2 μM RA, 0.25 μM Sant1, 500 nM PDBu, 200 nM LDN, Pancreatic Progenitor 1 stage – D8, D10, D12 – S3v3 supplemented with 10 mM Nicotinamide (Sigma, #N0636-100G), 50 ng/ml FGF7, 100 nM RA, 0.25 μM Sant1, 100 nM LDN. Pancreatic Progenitor 2 stage – D13, D15: S5 supplemented with 100 nM RA, 0.25 μM Sant1, 1 μM XXI, 10 μM Alk5iII, 1 μM T3, 20 ng/ml Betacellulin, 100 nM LDN. D17, D19: S5 supplemented with 100 nM RA, 0.25 μM Sant1, 1 μM XXI, 10 μM Alk5iII, 1 μM T3, 20 ng/ml Betacellulin, 100 nM LDN, 10 μM Isoxazole 9 (ISX9) Selleckchem, 57914). Endocrine Progenitor stage and SC-derived β cell stage – D20: ESFMv2 supplemented with 10 μM Alk5iII, 1 μM T3, 1 mM N-Actyl-L-cysteine (NAC) (Sigma Aldrich, #A7250), 10 μM ISX9. D22 – ESFMv2 supplemented with 10 μM Alk5iII, 1 μM T3, 1 mM NAC. D24 to D34: 10 μM Alk5iII, 1 μM T3, 1 mM NAC, 0.5 μM R428 (Selleckchem, S2841).

#### Dissociation and lentiviral transduction for shRNA knockdown studies

On D18, cells were rinsed with 1x PBS, and dissociated for eight min in the incubator with TrypLE™. Neutralization media comprising of 10% FBS in PBS was added, and cells passed through a 40 µm cell strainer. The cells were then centrifuged at 1200 rpm for 3 min. For planar culture, tissue culture-treated 24-well or 6-well plates were coated with coating media comprising of high glucose Dulbecco’s modified Eagle’s medium (DMEM) (Hyclone, SH30243.01) supplemented with 1% ECM (Sigma Aldrich, E1270) and 2 µg/mL fibronectin (Sigma Aldrich, F1141) for a minimum of ten min. Coating media was aspirated and cells were seeded. For reaggregation to spheroids, cells were seeded at a density of 4 million cells/well into non-treated 6-well plates and cultured on a shaker at 80 rpm. The next day, cells were transduced with lentiviruses at a multiplicity of infection (MOI) of 50 and 8 μg/ml of polybrene (Sigma Aldrich, TR-1003). Media was changed to S5 without growth factors the following day. Differentiation proceeded two days after viral transduction at D19. Selection for cells with successful lentiviral integration was carried out with blasticidin (InvivoGen, #ant-bl-1) on D22 to D26, at doses ranging from 2 μg/ml to 15 μg/ml.

#### EndoC-βH1 cell culture

Prior to cell culture, plates were coated with EndoC-βH1 coating media (DMEM with high glucose supplemented with 1% extracellular matrix (Sigma Aldrich, E1270) and 2 μg/ml fibronectin (Sigma Aldrich, F1141). EndoC-βH1 culture media consisted of DMEM with low glucose (Gibco, 11885084) supplemented with Bovine Serum Albumin (Sigma Aldrich, A9418), 2 mM L-glutamine (Gibco, 25030081), 50 μM 2-mercaptoethanol (Sigma Aldrich, M6250), 10 mM nicotinamide (Sigma Aldrich, N3376), 5.5 μg/ml transferrin (Sigma Aldrich, T13309) and 6.7 ng/ml sodium selenite (Sigma Aldrich, 214485).

#### Viral transduction in EndoC-βH1 cells

EndoC-βH1 cells were centrifuged with lentiviruses at a MOI of 50, for 55 min at 34 °C at 600 g. Cell pellet was then resuspended in the lentivirus-containing media and seeded in either 24-well plate format or on coverslips in a 12-well plate. Media was changed the following day.

#### 293FT cell culture

293FT cells were cultured in high glucose DMEM supplemented with 10% heat-inactivated fetal bovine serum (FBS) (Hyclone, 11521851) and 1% Non-Essential Amino Acids (NEAA) (Invitrogen,11140050). Cells were passaged weekly.

### Cloning of shRNA constructs into pLKO.1 vector

shRNA constructs targeting *SMAD9* were designed according to Genetic Perturbation Platform (Broad Institute). Primer sequences are listed in Supplementary Table 6. Primers were annealed and ligated into a pLKO.1 construct containing the blasticidin resistance gene cut with Age1 (NEB #R3552).and EcoRI (NEB #R3101) with the Quick Ligation™ Kit (New England Biolabs, M2200L). Successful ligation was confirmed with Sanger sequencing and plasmids were amplified for further experimental use.

### Lentivirus generation

Third generation lentiviruses were generated by transfecting confluent 293FT cells in 15 cm culture plates with DNA mixed with calcium phosphate bubbled into 2x HEPES buffered saline. shRNA pLKO constructs and viral packaging plasmids pRC/CMV-Rev (Rev), pHDM-HIVgpm (Gag/Pol) and pHDM-G (Vsv-g) were transfected at an amount of 24 ug, 1.2 ug, 1.2 ug, and 2.4 ug respectively per 15 cm plate. Media was changed to 293FT culture media 24 h after transfection, and the virus-containing media collected 48 and 72 h after transfection. Viruses were concentrated by passing through 0.45 μm filter and ultracentrifuging at 23,000 rpm for 2 h at 4 °C. Viruses were titred with the One-Wash Lentivirus Titer Kit (Origene, TR30038), and stored at -80 °C until use.

### qRT-PCR assay

Nucleospin™ RNA kit (Macherey-Nagel™,740995.250) was used to isolate total RNA from cells at various stages of β cell differentiation according to manufacturer’s protocol. Total RNA was reverse transcribed to cDNA using the High-Capacity cDNA Reverse Transcription Kit (Applied Biosystems), in an Applied Biosystems™ SimpliAmp™ Thermal Cycler (ThermoFisher Scientific), and diluted to a final concentration of 1 ng/μl. Each qPCR reaction consisted of 2.5 μl of cDNA, 5 μl of iTaq™ Universal SYBR® Green Supermix (Bio-Rad, L001752A), 0.3 μl of forward and reverse primer each (10 μM) and 1.9 μl of H_2_O to a final volume of 10 μl. The reactions were carried out in duplicates on a 384-well plate, and ran on the CFX384 Touch™ Real-Time PCR Detection System. The thermal cycling reaction was as follows: 95 °C for three min, followed by 40 cycles of 95 °C for 5 s, and 60 °C for 30 s. Primers were designed to span the exon–exon junction, and Ct values were normalized to *ACTIN* Ct values. Primer sequences are listed in Supplementary Table 6. The gene expression was analyzed accordingly to the 2^^-ΔΔCt^ method.

### SDS-PAGE and Western blotting assay

Cells were harvested by washing once with 1x PBS, followed by scraping in ice-cold protein lysis buffer consisting of 50 mM Tris-HCl, pH 8.5, 150 mM NaCl, 1% NP-40 (Merck, 492018), 1% protease inhibitor cocktail (Sigma Aldrich, P8340), 1% phosphatase-2 inhibitor (Sigma Aldrich, P5726), and 1% phosphatase-3 inhibitor (Sigma Aldrich, P0044). Protein samples were quantitated using the Pierce BCA Protein Assay Kit (Life Technologies, PI23227) and protein samples were boiled at 99 °C for five min, before being separated with SDS-PAGE for 120 V for 90 min. Samples were transferred to PVDF membranes and blocked for 1 h in 3% or 5% milk in TBST (1x Tris-Buffered Saline, 0.1% Tween 20) for 1 h. Membranes were incubated with primary antibodies diluted in milk overnight at 4 °C, followed by incubation for 1 h with secondary antibodies at room temperature. Membranes were then imaged with SuperSignal™ West Dura Extended Duration Substrate (Thermo Scientific, 34075) using an iBright CL750 Imaging System. The net ratio of SMAD9 levels over VINCULIN was quantitated with FIJI. Antibodies used are listed in Supplementary Table 6.

### Immunostaining analyses

EndoC-βH1 cells were transduced at an MOI of 50 and then seeded on coverslips at a density of 800,000 cells per well of a 12-well plate. Cells were washed once with PBS and fixed for 15 min with 4% paraformaldehyde, and blocked for 1 h at room temperature with blocking buffer (5% Bovine serum albumin in PBS with 0.2% triton X-100). Cells were then incubated with primary antibodies overnight at 4 °C. Cells were then washed once with PBS followed by incubation with secondary antibodies at room temperature in the dark for 1 h. Cells were washed thrice and then stained with DAPI for 10 min, followed by mounting with VECTASHIELD® Antifade Mounting Medium (H-1000-10). Confocal images were acquired with the use of Zeiss LSM 800 microscope. Images were analyzed with FIJI and mean fluorescent intensity (MFI) of each protein was normalized to that of DAPI. Antibodies used are listed in Supplementary Table 6.

### Flow cytometry analyses

hiPSC-derived β-like cells were dissociated by treatment with TrypLE™ for up to 10 min at 37 °C. Gentle pipetting with a P1000 pipette was carried out intermittently. Neutralization media comprising of 10% FBS in PBS was added, and cells passed through a 40 µm cell strainer. The cells were then centrifuged at 1200 rpm for 3 min and aliquoted into wells of a 96-well V-bottomed plate. The cells were fixed for 15 min with 4% paraformaldehyde, and blocked for 1 h at room temperature with FACS buffer comprising of 5% FBS in PBS with 0.1% Triton X-100. Primary antibody was added at a dilution of 1:100 for 1 h, followed by secondary antibody at 1:1000 dilution. For cell cycle flow cytometry, KI67 was used as the primary antibody followed by anti-rabbit Alexa fluor 594 as the secondary antibody. Cells were then stained with DAPI at 1:5000 for 15 min and followed by flow cytometry analysis with BD FACSymphony™ A3 Cell Analyzer (BDbiosciences). A minimum of 20,000 events were recorded per sample. Antibodies used are listed in Supplementary Table 6.

### Glucose-stimulated insulin secretion (GSIS) assay

EndoC-βH1 cells were seeded at a density of 300,000 cells/well in a 24-well plate after viral transduction with shRNA constructs targeting *SMAD9*. Cells were preincubated in an incubator for 1 h in Kreb’s Ringer Bicarbonate (KRB) Buffer (129 mM NaCl, 4.8 mM KCl, 2.5 mM CaCl_2_, 1.2 mM MgSO4, 1.2 mM KH_2_PO_4_, 5 mM NaHCO_3_, 10 mM HEPES, 0.1% BSA in ddH2O, pH 7.4 and sterile filtered) supplemented with 2.8 mM D-glucose. Supernatant was discarded, cells were washed twice with KRB and then incubated in KRB supplemented with 2.8 mM D-glucose for another hour. Supernatant was collected, cells were washed twice with KRB and incubated in KRB supplemented with 16.7 mM D-glucose for another hour. Supernatant was collected again, and the insulin secreted was quantitated with a human insulin ELISA kit (Mercodia, 36542). Total insulin content was obtained by overnight incubation of cells with 1 ml of acid ethanol solution. Cells were then centrifuged at 7000 rpm for 10 min and half of the supernatant was quantified for total insulin content. The other half of the supernatant was boiled to a dry pellet and resuspended in nuclease-free water, and genomic DNA levels were quantified with a nanodrop.

### Statistical analyses

All statistical analyses were conducted with GraphPad Prism. One-way ANOVA was used with Tukey’s post hoc test for groups without an assigned control (e.g., qRT-PCR for gene expression across four time points) or Dunnett’s post hoc test for groups with a defined control (e.g., knockdown experiments). Two-way ANOVA with Sidak’s post hoc test was performed for data involving multiple groups. Statistical significance was defined as *P* ≤ 0.05.

## Discussion

While the transcriptomic profiles of both pancreatic islets and hPSC-derived pancreatic cells have been intensely studied, with a focus on the early development of pancreatic progenitors or the comparison of hPSC-derived β-like cells with adult human islet cells [3, 13–15, 17, 22, 33–35]. The transition of human pancreatic endocrine progenitors to β-like cells remains less well understood. Here, we comprehensively analyzed the transcriptomes of hESC- and hiPSC-derived D20 pancreatic endocrine progenitors and D35 β-like cells to elucidate genes and transcription factors that follow uniquely conserved expression patterns during human pancreatic β cell maturation.

In particular, the group of genes found to be increasingly upregulated from D20 to D35 are involved in response to stimulus, hormone transport and regulation of hormone levels, suggesting endocrine hormonal function. This is consistent with increased maturation of D20 pancreatic endocrine progenitors toward D35 hormone-expressing endocrine cells. Reciprocally, the genes that are decreasing in expression are involved in nervous system and neuronal development, indicating the downregulation of neural program in favor of that of the endocrine. This corroborates the findings of Ohta et al., who reported that despite being derived from different germ layers, pancreatic islet cells and neurons have interrelated transcriptional cascades [36]. Our current findings also imply the importance of developmental transition and trajectory from D20 endocrine progenitors toward maturing human β cells.

Another key observation from our detailed RNA-Seq analyses is the yet-unreported downregulation of BPs related to cell cycle, such as chromosome segregation, mitotic cell cycle and nuclear division, in D20 and D35 pancreatic cells. The mitotic kinesin motor protein KIF14 localizes to the spindle during metaphase, then to the midzone in anaphase and then to the midbody during cytokinesis [37, 38]. E2F7 is involved in DNA replication and repair [39, 40]. It is targeted by APC/CCdh1 during the G_1_ phase for degradation and undergoes proteasomal degradation during the G_2_ phase, after being targeted by SCFcyclin F [39, 41]. SKA1 and SKA3 make up part of the three-subunit complexes (Ska1-Ska2-Ska3) that are critical microtubule-binding factors which maintain load-bearing kinetochore-microtubule attachments; and function to recruit protein phosphatase1 (PP1) to kinetochores [42]. After being recruited to the kinetochore, PP1 functions to dephosphorylate kinetochore proteins, thus opposing spindle checkpoint signaling and promoting the onset of anaphase [43, 44]. Lastly, *MKI67* encodes for KI67, which is required for the formation of the perichromosomal layer (PCL) during chromosomal condensation in mitosis and stays attached to it until telophase [45]. The presence of KI67 on the PCL functions to maintain proper distribution of nucleolar components during mitosis and also prevents the aggregation of mitotic chromosomes [46, 47]. The decrease in transcript and protein expression of these cell cycle genes in D20 and D35 pancreatic cells indicates lower mitotic activity that is consistent with β cell maturation.

While existing literature has reported mature pancreatic β cells to have decreased cellular proliferation [48, 49]. Our observations here are specific to hPSC-derived pancreatic endocrine progenitors progressing toward mature β-like cells, revealing the specific cell cycle genes and processes that are involved in this important cellular maturation. The decrease in proliferation as endocrine progenitors mature during human β cell differentiation is consistently observed but not formally reported before. Here, we specifically identified that the majority of D0 hPSCs were in the G_1_ phase of cell division, whereas D13, D20, and D35 pancreatic cells had exited the cell cycle and were mostly in the G_0_ phase, with an increasing percentage as differentiation progressed. This is analogous to that of bona fide human β cells, where a decline in proliferative capacity is observed as glucose responsivity increases during development [50, 51]. The reduced cell proliferation observed during pancreatic differentiation in this study mirrors the developmental process, where the transition from primitive to specialized cells is characterized by a gradual decline in proliferative capacity, ultimately leading to cell cycle exit [52]. Our findings are in agreement to that of Ameri et al., who identified negative cell cycle regulators CDKN1A (p21) and CDKN2A (p16) to be enriched in PDX1^+^NKX6.1^+^ pancreatic endocrine cells at day 17 [53]. While Ameri et al. looked at the regulation of the cell cycle during differentiation, we report and characterize the general decrease in cell cycle activity towards β-like cells. Our flow cytometry analyses on the decrease in cell cycle activity during differentiation complement the findings of Kim et al., who reported a similar trend in the decrease of proliferative cells derived from mouse embryos [54]. Various groups have employed cell cycle inhibitors such as ZM447439 [15] and CDK inhibitors [55] in the later stages of differentiation, further suggesting that decreasing cell cycle activity might be critical for proper transition towards fully differentiated β cells.

Amongst the many newly-identified genes and transcription factors upregulated in D20 pancreatic endocrine progenitors and D35 β-like cells, we studied the biology of SMAD9 in greater detail, with the aim of identifying a new transcription factor that might be involved in the specification of endocrine progenitors to β-like cells. Currently, there is limited literature on the specific function that SMAD9 may play in human β cells. Schreiber et al. (2021) demonstrated using CUT&RUN on pancreatic endocrine progenitors derived from a NEUROG3-tagged hiPSC line that *SMAD9* is one out of 1,263 targets of NEUROG3 [56]. *SMAD9* expression can be found to be enriched in early, late stem cell (SC)-derived β cells and in adult β cells [15, 17], with strong positive association with β but not α cells [30]. Additionally, *SMAD9* can be identified as enriched in β cells via deep sequencing of the human pancreas with SORT-seq, but not in the other islet cell types [57]. *SMAD9* has also recently been found to be downregulated in T2D compared to healthy islets via RNA-Seq analyses [58]. Furthermore, RNA-Seq analyses of 0.5 mM Palmitate Washout Islets (D8) versus 0.5 mM Palmitate-Exposed Islets (D4) identified *SMAD9* to be amongst 395 genes to be upregulated in human islets recovering from palmitate treatment [59]. These findings imply the involvement of SMAD9 in human β cell insulin secretion function.

Knockdown of *SMAD9* significantly reduced β cell markers in D35 β cells and EndoC-βH1 cells. On the other hand, while EndoC-βH1 cells had reduced insulin secretion, total insulin content in these cells remained unchanged. One possible explanation is that SMAD9 plays a greater role in β cell development than in the maintenance of mature β cell function. This is in agreement with our findings from the analysis of Balboa et al.’s scRNA-Seq dataset [15], where we observed that *SMAD9* expression peaks in early sc-β cells and declines further during maturation. In addition, while the knockdown of SMAD9 was observed, the slow replication rate of EndoC-βH1 cells [60] might make the phenotypic consequences of *SMAD9* knockdown less obvious.

SMAD9, which is also known as SMAD8 or SMAD8/9 is a type of R-SMAD (Receptor-regulated SMAD). SMAD9 is known to be structurally similar to the other R-SMADs, SMAD1 and SMAD5, but the distinct roles the different R-SMADs play during development have yet to be elucidated [61]. SMADs are signaling effectors of the TGF-β family receptors [62]. These proteins are able to undergo nucleocytoplasmic shuttling, whereby R-SMADs are situated in the cytoplasm, and are phosphorylated by type-1 receptor kinases, then oligomerize with co-SMADs (SMAD4) and translocate to the nucleus to regulate gene transcription [61, 63, 64].

The expression of *SMAD2* appears high at D0 compared to the other stages of differentiation, likely due to activation by Activin A at the early stages of differentiation [10, 17, 23, 65–67]. which signals through SMAD2 [68, 69]. The expression of *SMAD2* and *SMAD3* appears to remain low after D13 onwards, due to the addition of Alk5ii inhibitor (ALX-270-445-M001) from D20 endocrine progenitor stage [10, 17, 23, 65–67]. which inhibits TGFβ type I receptor that regulates SMAD2 and SMAD3 [70]. While mass spectrometry analyses (as compared to RNA-Seq) are more appropriate to study the levels of BMP ligands that may activate the SMADs, our time course analyses of BMP ligands show a contrasting expression throughout differentiation to that of *SMAD9*. The majority of the BMP ligands were identified to have greater expression at D0 compared to the other timepoints, whereby BMP signaling is critical in directing pluripotent cells to extraembryonic fates [71]. This agrees with the higher transcript expression of the other SMADs at early stages of differentiation. However, deeper studies involving proteomic analysis are required before such premature conclusions can be made.

There are a few studies investigating the signaling mechanisms of SMAD9. Tsukamoto et al. proposed that SMAD9 is a new type of transcriptional regulator in BMP signaling [72]. Co-immunoprecipitation assays revealed that SMAD9 interacts with SMAD1 (another R-SMAD) and SMAD4 (co-SMAD), and the overexpression of chimeric SMAD9 with either the MH1 domain, linker region or MH2 domain of SMAD9 substituted, followed by luciferase assays showed that the linker region of SMAD9 suppresses the transcriptional activity of other SMADs [72]. The authors further suggest that SMAD9 is a dominant negative SMAD, as it forms complexes with BMP-regulated R-SMADs and binds to BRE (BMP response element) [72]. Tsukamoto et al. conducted these studies on mouse myoblasts [72], whereby BMP signaling plays a critical role in regulating cell fate by promoting osteogenic differentiation and inhibiting myogenic differentiation [73, 74]. The postulation that SMAD9 is a dominant negative SMAD to the other R-SMADs supports our observations where SMAD9 expression peaks during endocrine progenitor differentiation towards β-like cell stage, opposing the trends of *SMAD1* and *SMAD5*, which decrease in expression from D20 onwards. scRNA-Seq analysis of different cell types during β cell differentiation shows an opposing trend of BMP ligands and *SMAD9*, with *BMP2*, *BMP4*, *BMP7* and *GDF7* being highly expressed in non-endocrine cells, unlike *SMAD9* [17].

Dekker et al. demonstrated that the activation of BMP signaling through the addition of recombinant BMP2 or BMP4 (which activates R-SMADs (SMAD1, SMAD5) altered β cell identity, with reduced MAFA and INS levels alongside impaired GSIS in INS-1E cells [75]. The authors reported increased HES1 expression in human islets [75]. SMAD9 was also reported to be mainly found in β cells [75]. These findings strongly suggest that enhanced BMP signaling is detrimental to β cell function. In line with the earlier reported literature suggesting that SMAD9 is a dominant negative SMAD to the other R-SMADs, these findings support ours, where we show that SMAD9 is important for the differentiation of endocrine progenitors to β cells. Taking the aforementioned literature into account, we then postulate that SMAD9 might serve as a dominant negative SMAD, opposing the negative effects of BMP signaling through BMP type-1 receptor R-SMADs SMAD1 and SMAD5, to allow for proper β differentiation in endocrine progenitors and also to maintain β cell identity in mature β cells.

Overall, our in-depth studies have now demonstrated a clear positive role for SMAD9 during human pancreatic endocrine progenitor differentiation toward maturing hPSC-derived β-like cells, regulating key β cell markers such as *PDX1*, *NKX6.1*, and insulin content. Our RNA-Seq analyses further revealed important downstream β cell targets of SMAD9, including *GLIS3*, *MAFA*, *HNF1B*, *NEUROD1*, *NKX2.2* and *ABCC8*. In mature EndoC-βH1 human β cells, we also found SMAD9 to be necessary for GSIS function.

In conclusion, we have comprehensively analyzed RNA-Seq data from hESC- and hiPSC-derived pancreatic β-like cells, revealing common genes and transcription factors that are uniquely up- or downregulated temporally. We characterized the decrease in cell proliferation observed during D20 β cell differentiation. Additionally, we identified and studied a new transcription factor, SMAD9, upregulated on D20 and D35 of β cell differentiation to play an important role in β cell identity and function. Our work provides an invaluable resource of new genes and transcription factors that could be intimately involved in driving the important transition from human pancreatic endocrine progenitors, maturing toward β-like cells.

### Limitations of the study

To knockdown *SMAD9* specifically at the pancreatic endocrine progenitor stage, we had to dissociate our pancreatic “organoids” at D18, transduce single cells with shRNA lentiviral constructs targeting *SMAD9*, before reaggregating them in suspension culture and differentiating them till D35 β-like cells. This resulted in excessive cell loss, limiting and affecting the usual suspension differentiation of β-like cells. Therefore, we were unable to perform GSIS on the hPSC-derived D35 β-like cells and had to rely on EndoC-βH1 cells for functional assays. While we were able to perform mechanistic studies on SMAD9 biology, including RNA-Seq analyses on shSMAD9 β cells, much of this manuscript focused on the use of this differentiation platform to identify genes and transcription factors involved in the transition from endocrine progenitors to β-like cells. Therefore, the identification of SMAD9-bound targets or SMAD9 signaling is currently out of scope of this manuscript.

EndoC-βH1 cell line is now a routinely used immortalized human pancreatic β cell line, demonstrated by the β cell community to respond to high glucose and secrete insulin fairly reproducibly [76]. However, hPSC-derived β-like cells are known to exhibit variable glucose-responsiveness due to the genetic background of the cell line and differences in the ability of the line to differentiate into mature glucose-responsive β-like cells [15–17]. In general, several groups have described the need for further in vivo maturation, at times, in order for these β-like cells to elicit glucose-responsiveness reproducibly [15, 16, 66, 67]. Therefore, a part of this work leveraged EndoC-βH1 cells to study the consequence of knocking down *SMAD9* on the impact of β cell insulin secretion.

### Materials availability

Reagents generated in this study will be made available on request.

## Supplementary information

Supplementary Information

Suppl Figures

Table S1

Table S2

Table S3

Table S4

Table S5

Table S6

## Data Availability

RNA-Seq data have been deposited under SRA submission: SUB15340027, BioProject: PRJNA1267818.

## Code availability

RNA-Seq data have been deposited under SRA submission: SUB15340027, BioProject: PRJNA1267818.
