# Supplementary material for: RNA-seq at different stages of human pancreatic β cell differentiation reveals proliferation dynamics and SMAD9 in directing β cell fate
Source: Cell Death Dis. 2026 Mar 10;17(1):302. doi: 10.1038/s41419-026-08529-z (PMC13039864; doi:10.1038/s41419-026-08529-z)
Supplement: Supplementary file 1 — Supplementary Information [file 41419_2026_8529_MOESM1_ESM.docx]

**SUPPLEMENTARY INFORMATION**

**SUPPLEMENTARY FIGURE LEGENDS**

**Supplementary Figure 1. Detailed schematic of the filtering and sorting criteria for the identification of genes at various timepoints in both hESC and hiPSC differentiation into pancreatic β-like cells.**

**Supplementary Figure 2. Analyses of genes upregulated on D20 and D35 in hPSC differentiation into pancreatic β-like cells.** A) Detailed schematic of the filtering and sorting criteria for the identification of genes upregulated on D20 and D35 in both hESC and hiPSC differentiation into pancreatic β-like cells. B) Table detailing the exact number of genes falling into each category for the analyses performed in A). GO BP analyses for genes upregulated in D20 and C) near constant, D) increasing and E) decreasing at D35 in hPSC differentiation into pancreatic β-like cells.

**Supplementary Figure 3. Analyses of genes downregulated on D20 and D35 in hPSC differentiation into pancreatic β-like cells.** A) Detailed schematic of the filtering and sorting criteria for the identification of genes downregulated on D20 and D35 in both hESC and hiPSC differentiation into pancreatic β-like cells. GO BP analyses for genes downregulated in B) D20 and D35, and C) D20 only in hPSC differentiation into pancreatic β-like cells.

**Supplementary Figure 4. Characterization of cell cycle genes in hPSC differentiation into pancreatic β-like cells.** A) qRT-PCR analyses of cell cycle genes that are downregulated in D20 and D35 during hESC and hiPSC differentiation into pancreatic β-like cells. Results are from n=3 independent experiments. Statistical analyses were conducted with one-way ANOVA with Tukey’s post hoc test and considered to be statistically significant when P ≤ 0.05. B) Flow cytometry gating strategy used. C) Cell cycle flow cytometry gating at D0, D13, D20 and D35 of hiPSC differentiation into pancreatic β-like cells. One representative experiment is presented here. For all statistical analyses: Error bars represent standard deviation (STDEV).

**Supplementary Figure 5. Analyses of transcription factors differentially expressed in D20 and D35 in hPSC differentiation into pancreatic β-like cells.** A) Detailed schematic of the filtering and sorting criteria and B) summary table for the identification of transcription factors up- or downregulated on D20 and D35 in both hESC and hiPSC differentiation into pancreatic β-like cells. GO BP analyses for transcription factors downregulated in C) D20 and D35, and D) D20 only in hPSC differentiation into pancreatic β-like cells.

**Supplementary Figure 6. Analyses of SMAD9 during hPSC differentiation into pancreatic β-like cells.** A) Fold change of *SMAD9* at D0 v D20 and D0 v D35, for hESC- and hiPSC-derived β-like cells. P values are presented. B) shRNA constructs targeting *SMAD9*. C) Flow cytometry analyses of PDX1, INS and NKX6.1 in D35 β-like cells, where *SMAD9* is knocked down from D20. One representative experiment is presented here. D) PCA plot for shSCR and shSMAD9-1, -2 and -3. PC1: 83% variance, PC2: 9% variance. E) Box plot showing *SMAD9* transcript expression as assessed by bulk RNA-Seq in D35 β-like cells, where *SMAD9* is knocked down from D20. F) GO BP analyses for genes downregulated in D35 β-like cells, where *SMAD9* is knocked down from D20. G) Heatmap of *SMAD*s across the timepoints of hPSC differentiation into pancreatic β-like cells. H) Heatmap of BMP ligand genes across the timepoints of hPSC differentiation into pancreatic β-like cells.

**Supplementary Figure 7. Expression of *SMAD9* in human islets and hPSC-derived β-like cells.** *SMAD9* transcript levels as assessed by bulk RNA-Seq from humanislets.com, showing positive association with A) increasing insulin secretion at 16.7 mM glucose, B) increasing β cell proportion amongst endocrine cells and C) negative association with increasing α cell proportion amongst endocrine cells. D) Comparison of *SMAD9* expression across various cell clusters as assessed by single cell RNA-Seq performed by Balboa et al,, 2022. Results obtained from <https://singlecell.broadinstitute.org/single_cell/study/SCP1526> (15). E) UMAP plot of endocrine lineage towards early and then late sc-β cells as analyzed through Slingshot, with data from Balboa et al., 2022. F) *SMAD9* expression across the pseudotime trajectory analysis of endocrine progenitors, early and late sc-β cells; fold change of *SMAD9* expression in endocrine progenitors and early sc-β cells; and the fold change of *SMAD9* expression in early, late and adult β cells.

**SUPPLEMENTARY TABLE LEGENDS**

Supplementary Table 1. Differentially expressed genes at D20 or D35 in hESCs and hiPSCs differentiating into pancreatic β-like cells.

Supplementary Table 2. Genes uniquely upregulated in hPSC-derived a) D20 pancreatic endocrine progenitors or b) D35 β-like cells only and their associated GO BP.

Supplementary Table 3. Genes expressing a) near-constant, b) increasing or c) decreasing expression in hPSC-derived D20 to D35 pancreatic cells and their associated GO BP.

Supplementary Table 4. Genes a) consistently downregulated in both D20 and D35, b) in D20 only or c) D35 hPSC-derived pancreatic cells and their associated GO BP.

Supplementary Table 5. Differentially expressed genes at D35 upon SMAD9 knockdown in hiPSC-derived endocrine progenitors differentiating into pancreatic β-like cells.

Supplementary Table 6. List of antibodies and primer sequences used in this study.
