# Supplementary material for: RNA-seq at different stages of human pancreatic β cell differentiation reveals proliferation dynamics and SMAD9 in directing β cell fate
Source: Cell Death Dis. 2026 Mar 10;17(1):302. doi: 10.1038/s41419-026-08529-z (PMC13039864; doi:10.1038/s41419-026-08529-z)

Supplementary Figure 1: Lim et al.,

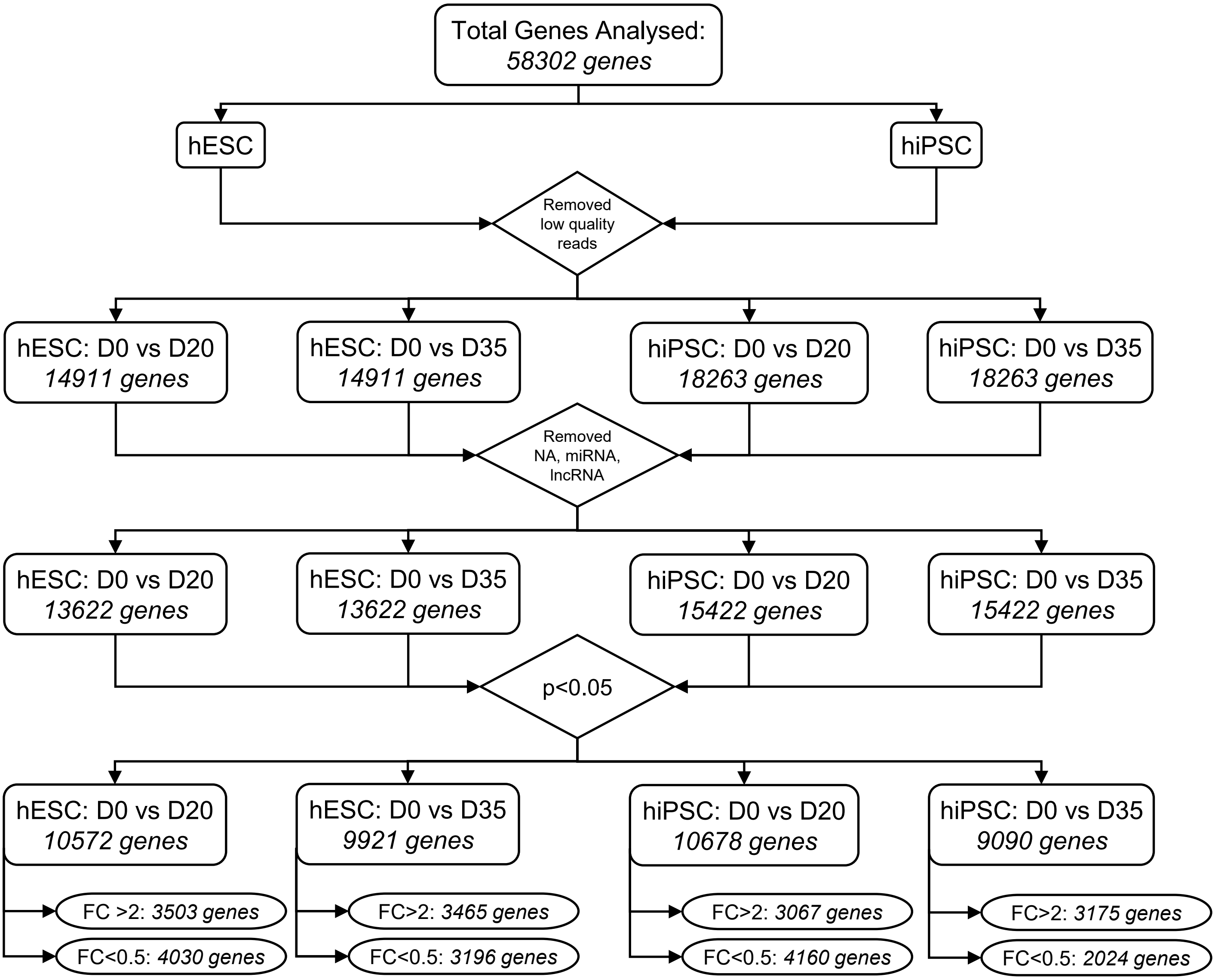

Supplementary Figure 2: Lim et al.,

A

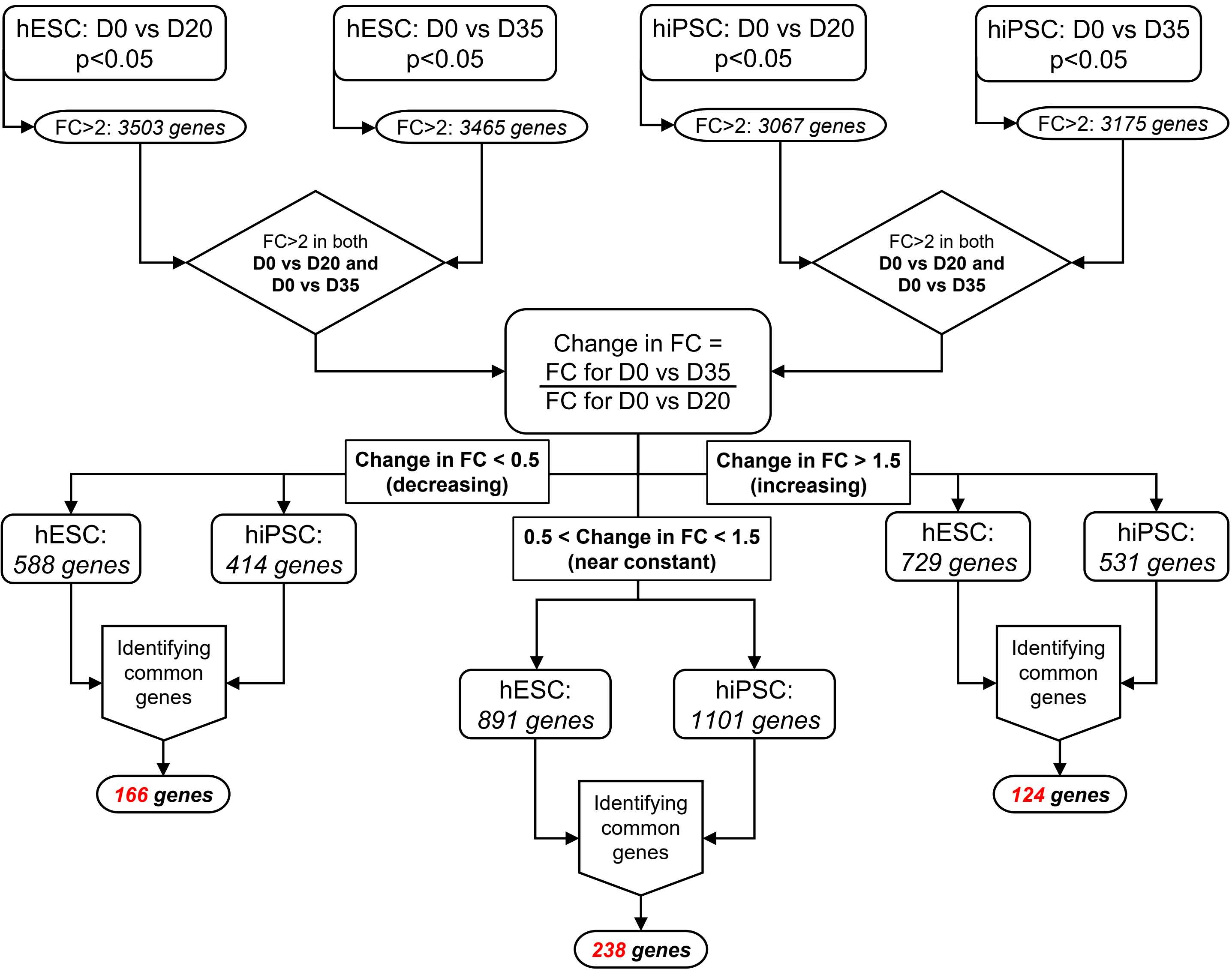

B

	Downregulated genes		
	D20 only	D35 only	Both
hESC	1636	802	2394
hiPSC	2834	698	1326

			Upregulated genes			
	D20 only	D35 only	Both	Both, Change in 0.5<FC<1.5 (no change)	Both, Change in FC >1.5 (increasing)	Both, Change in FC< 0.5 (decreasing)
hESC	1295	1257	2208	891	729	588
hiPSC	1021	1129	2046	1101	531	414

Supplementary Figure 2: Lim et al.,

C

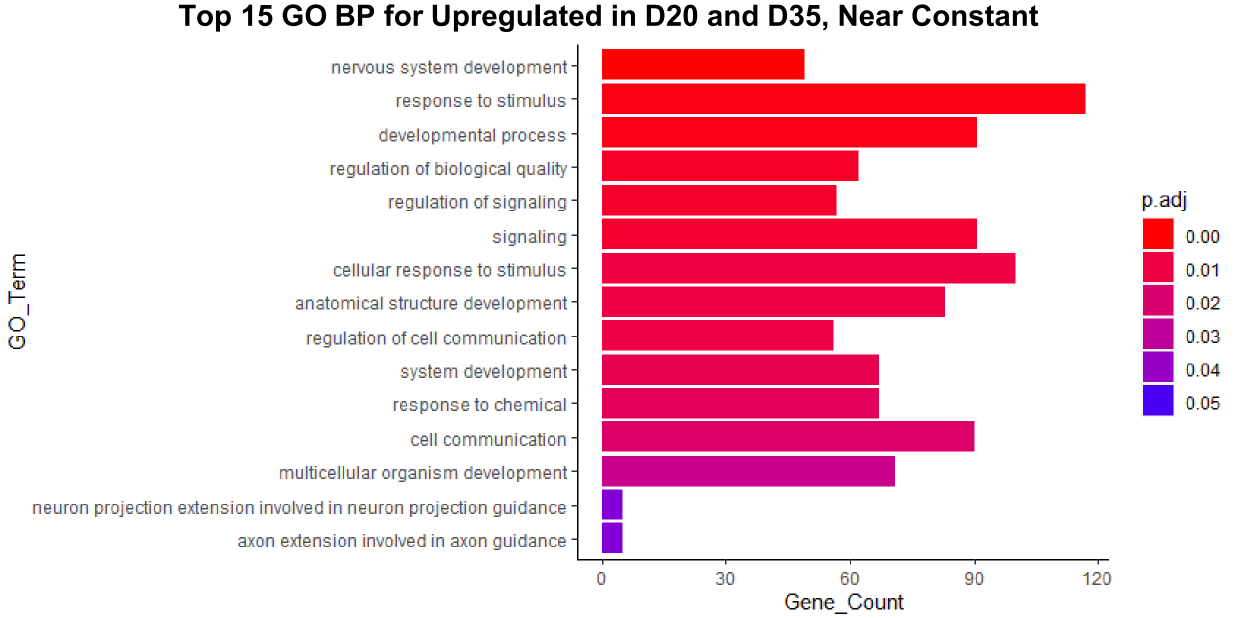

D

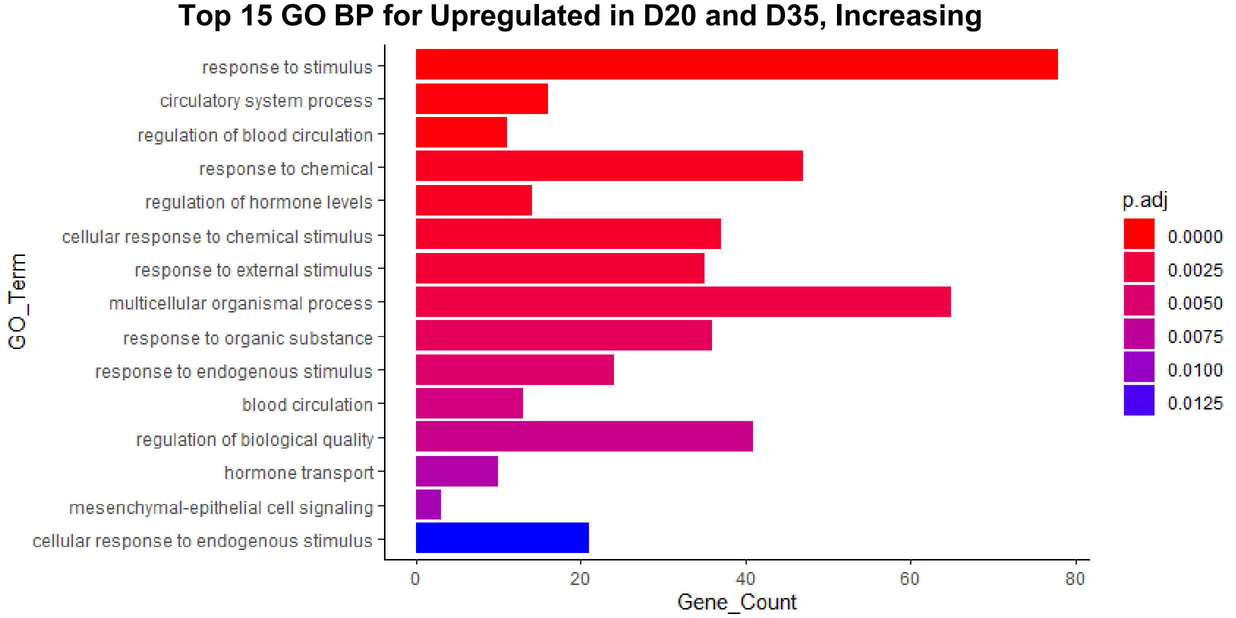

E

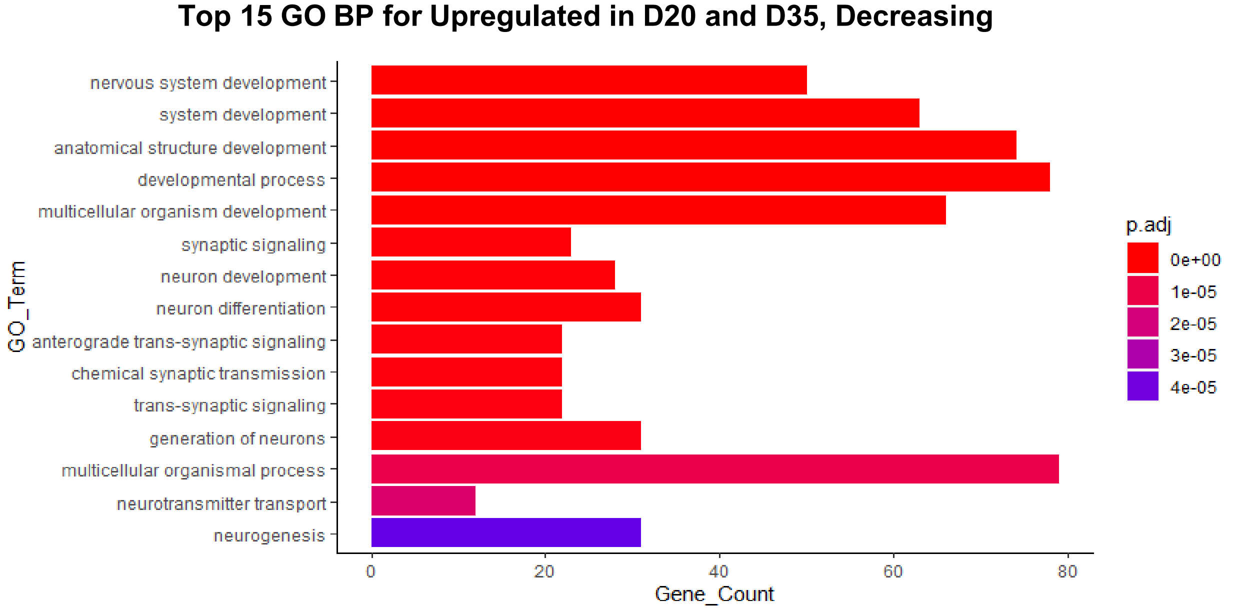

Supplementary Figure 3: Lim et al.,

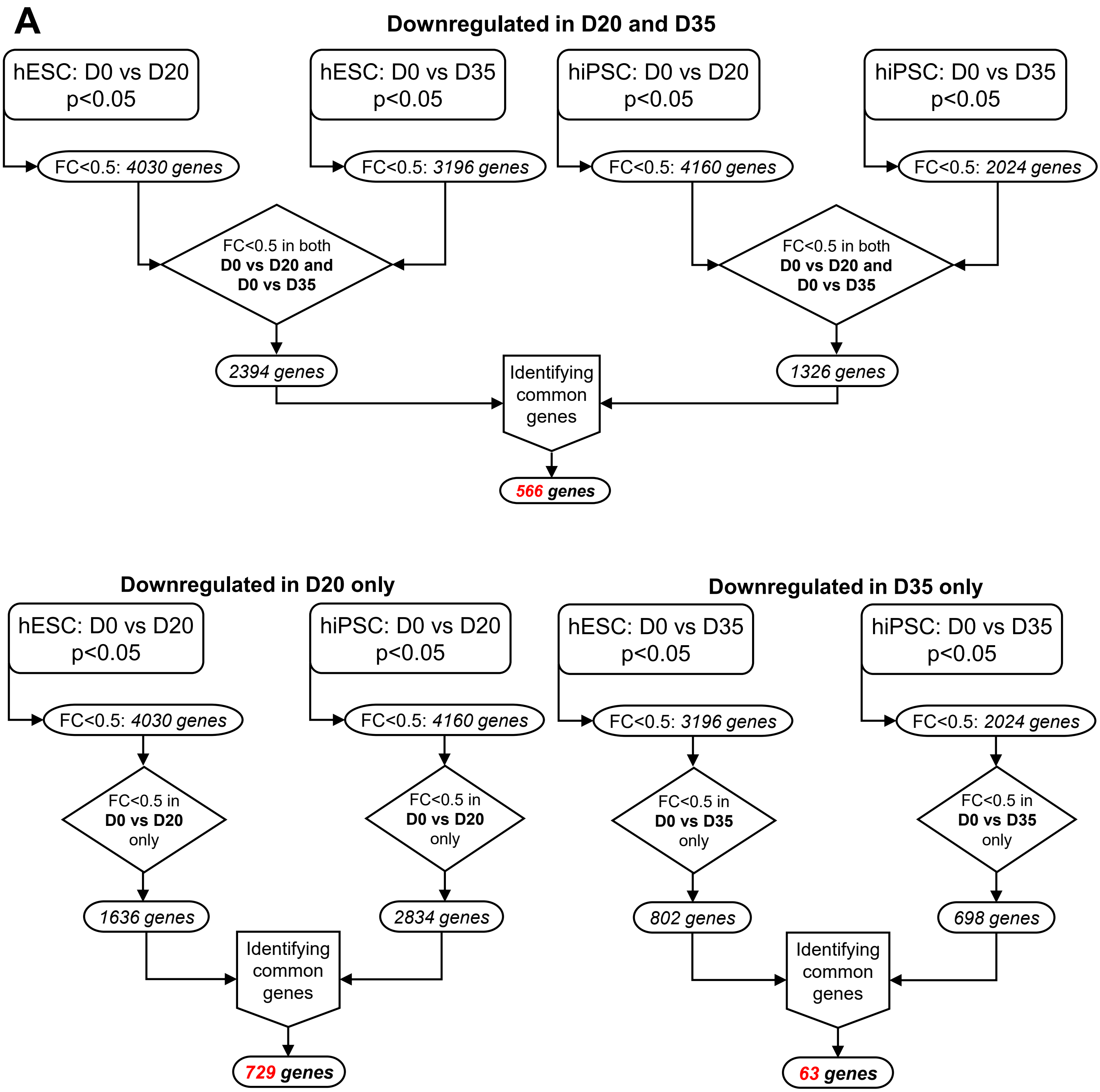

Supplementary Figure 3: Lim et al.,

B

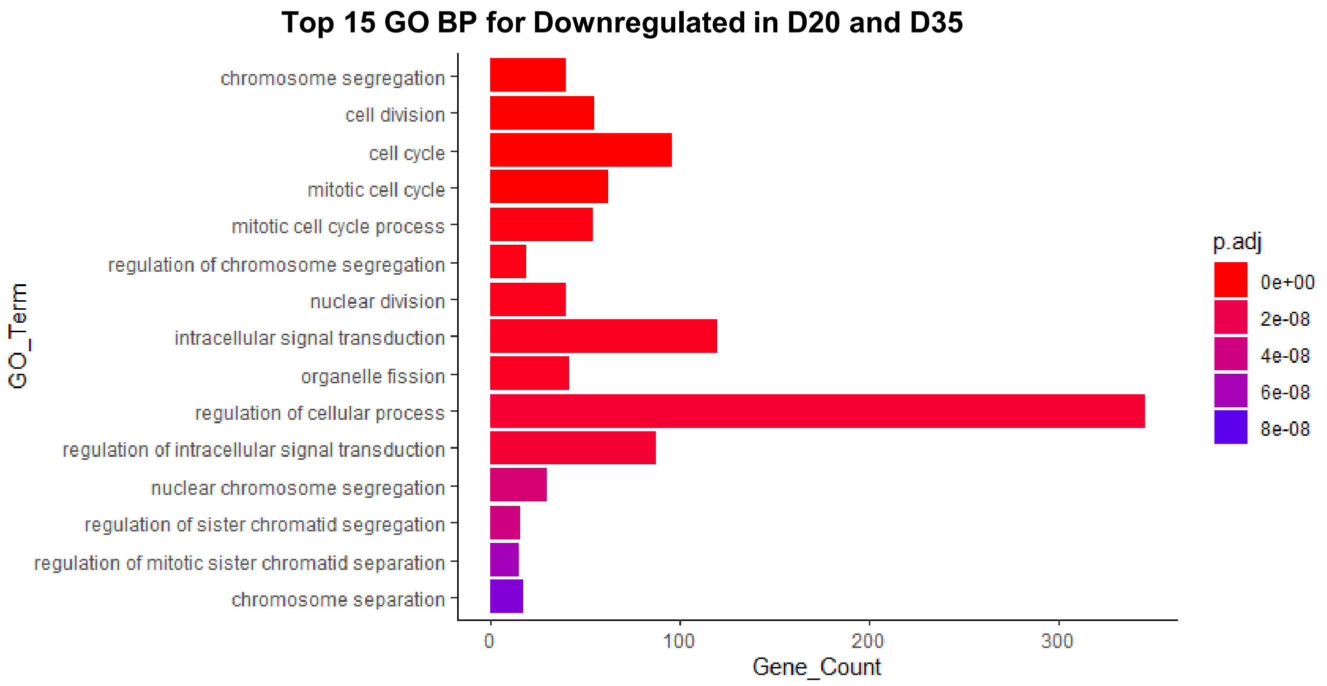

C

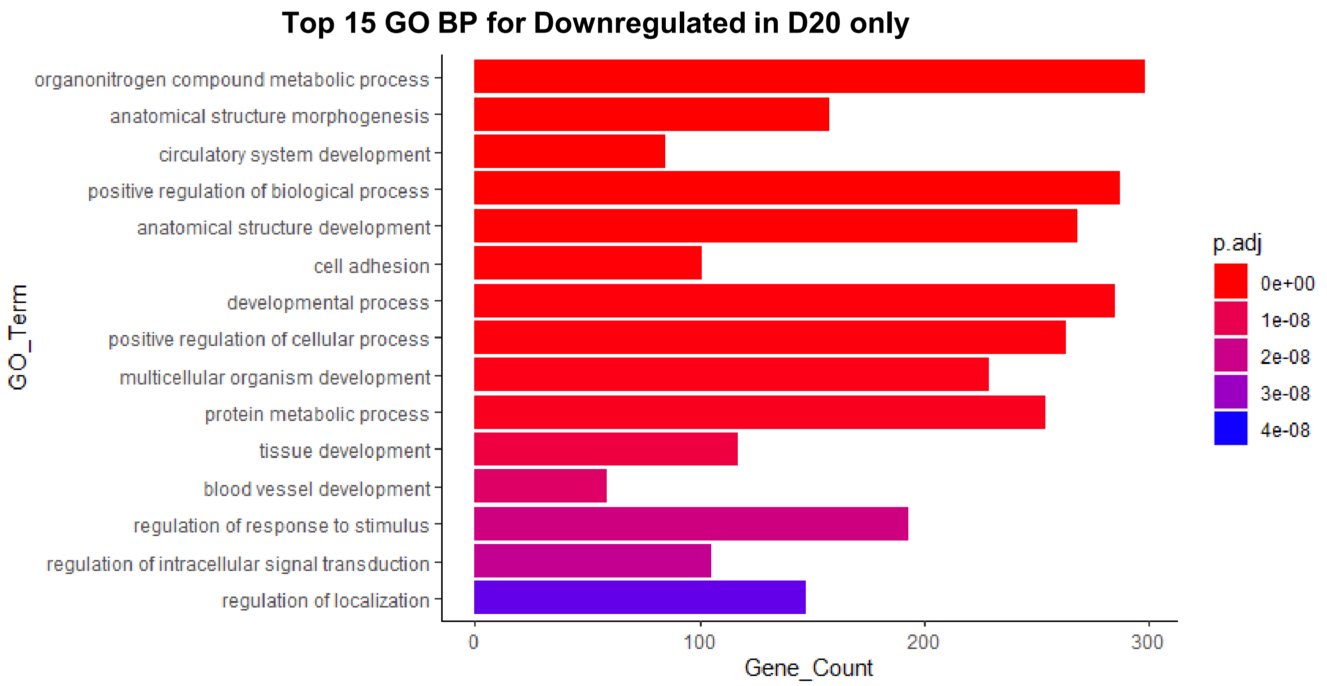

Supplementary Figure 4: Lim et al.,

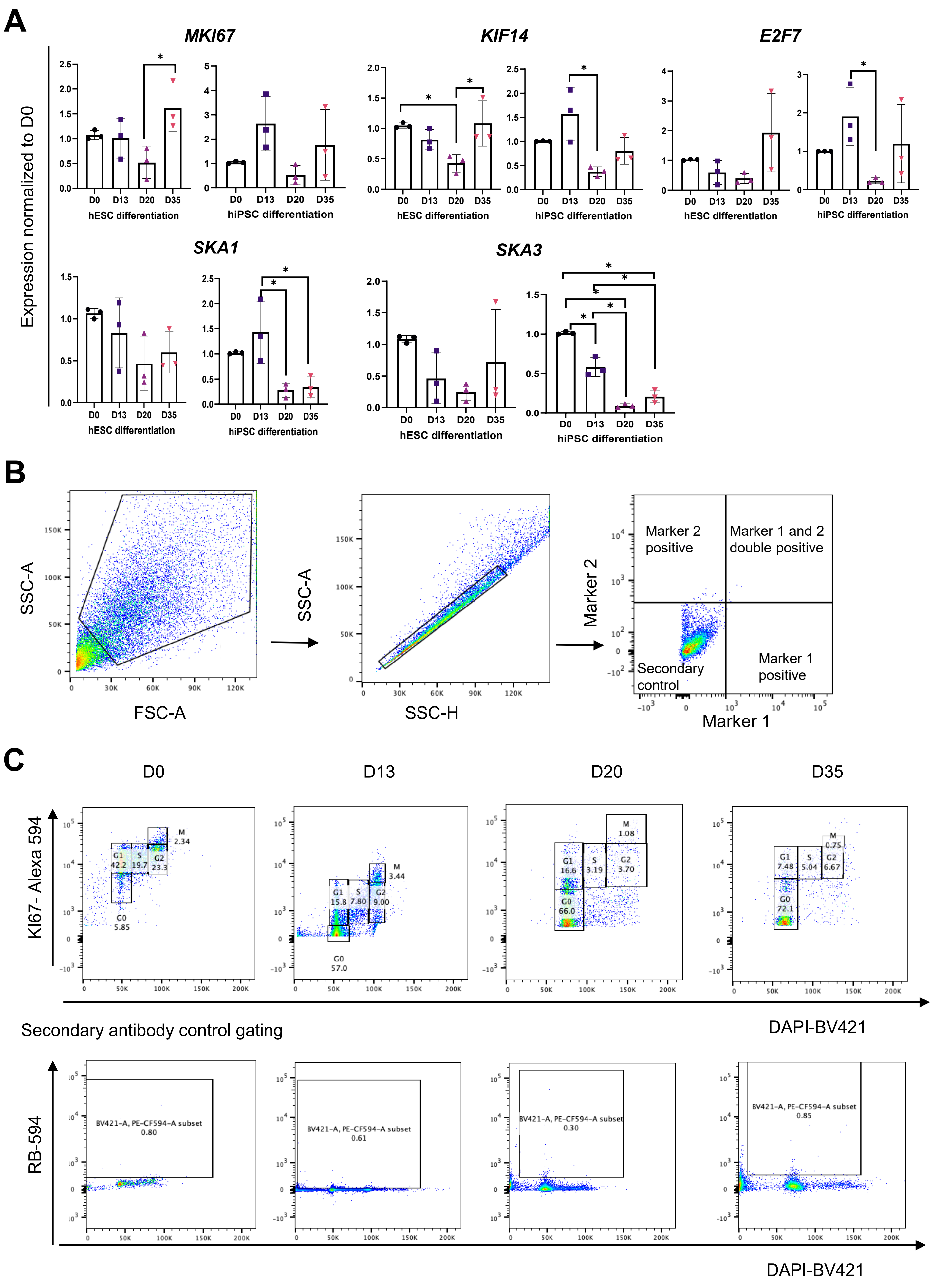

Supplementary Figure 5: Lim et al.,

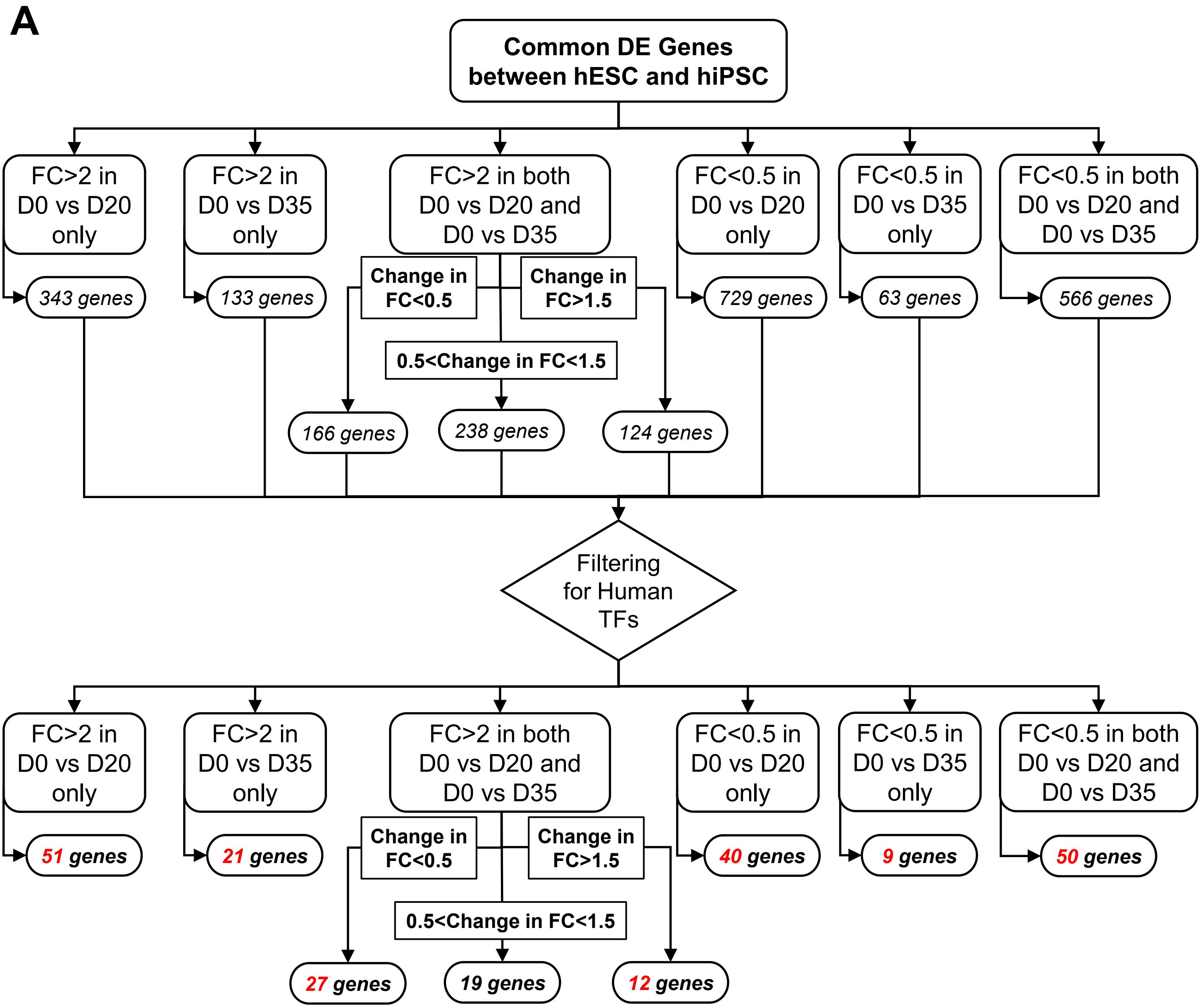

B

**Category**	**Total number of genes**	**Number of genes that are TFs**	**Percentage of genes that are TFs**
Upregulated in D20 only	343	51	14.87
Upregulated in D35 only	133	21	15.79
Upregulated in D20 and D35, Near Constant	238	19	7.98
Upregulated in D20 and D35, Increasing	124	12	9.68
Upregulated in D20 and D35, Decreasing	166	27	16.27
Downregulated in D20 and D35	566	50	8.83
Downregulated in D20 only	729	40	5.49
Downregulated in D35 only	63	9	14.29

Supplementary Figure 5: Lim et al.,

C Top 15 GO BP for TFs Downregulated in D20 and D35

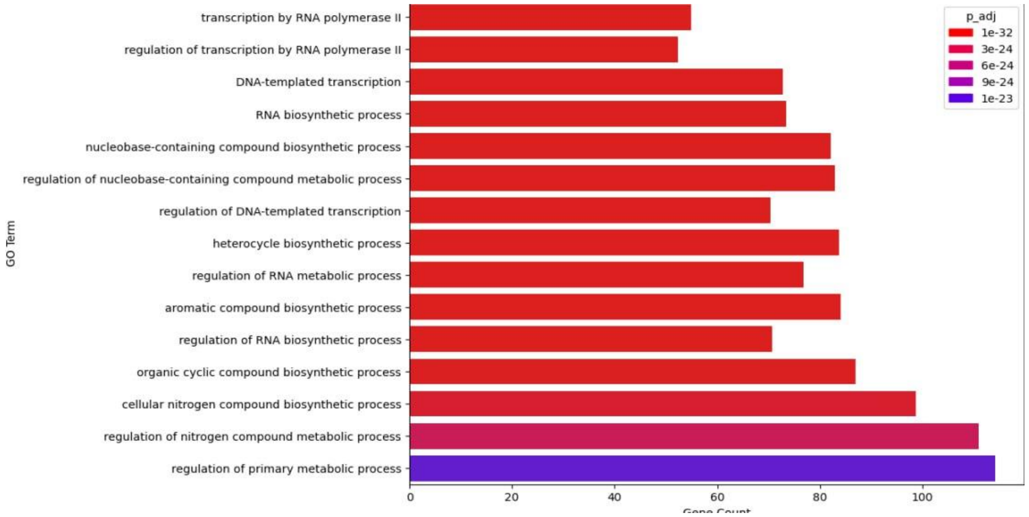

D Top 15 GO BP for TFs Downregulated in D20 only

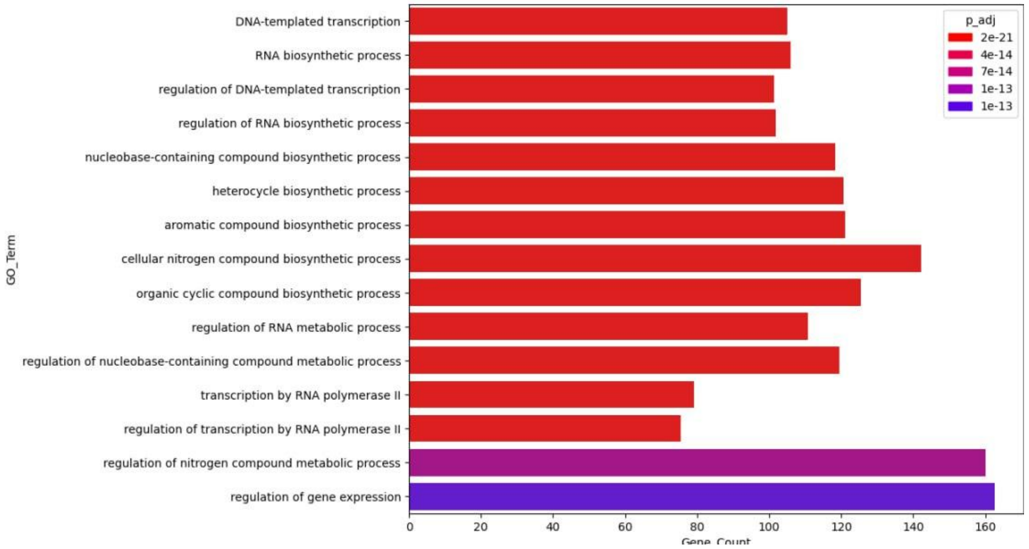

Supplementary Figure 6: Lim et al.,

A

SMAD9	FC (D0 v D20)	FC (D0 v D35)	P value (D0 v D35)
hESC	6.28	32.22829	1.60E-17
hiPSC	2.77978	9.72868	8.16E-29

B

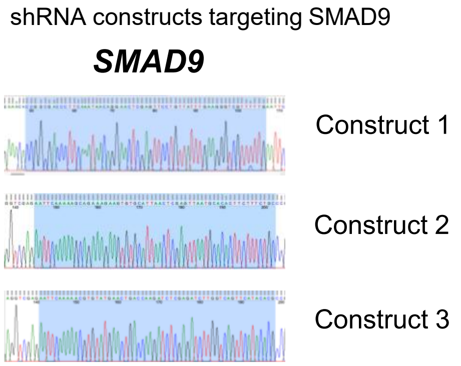

C

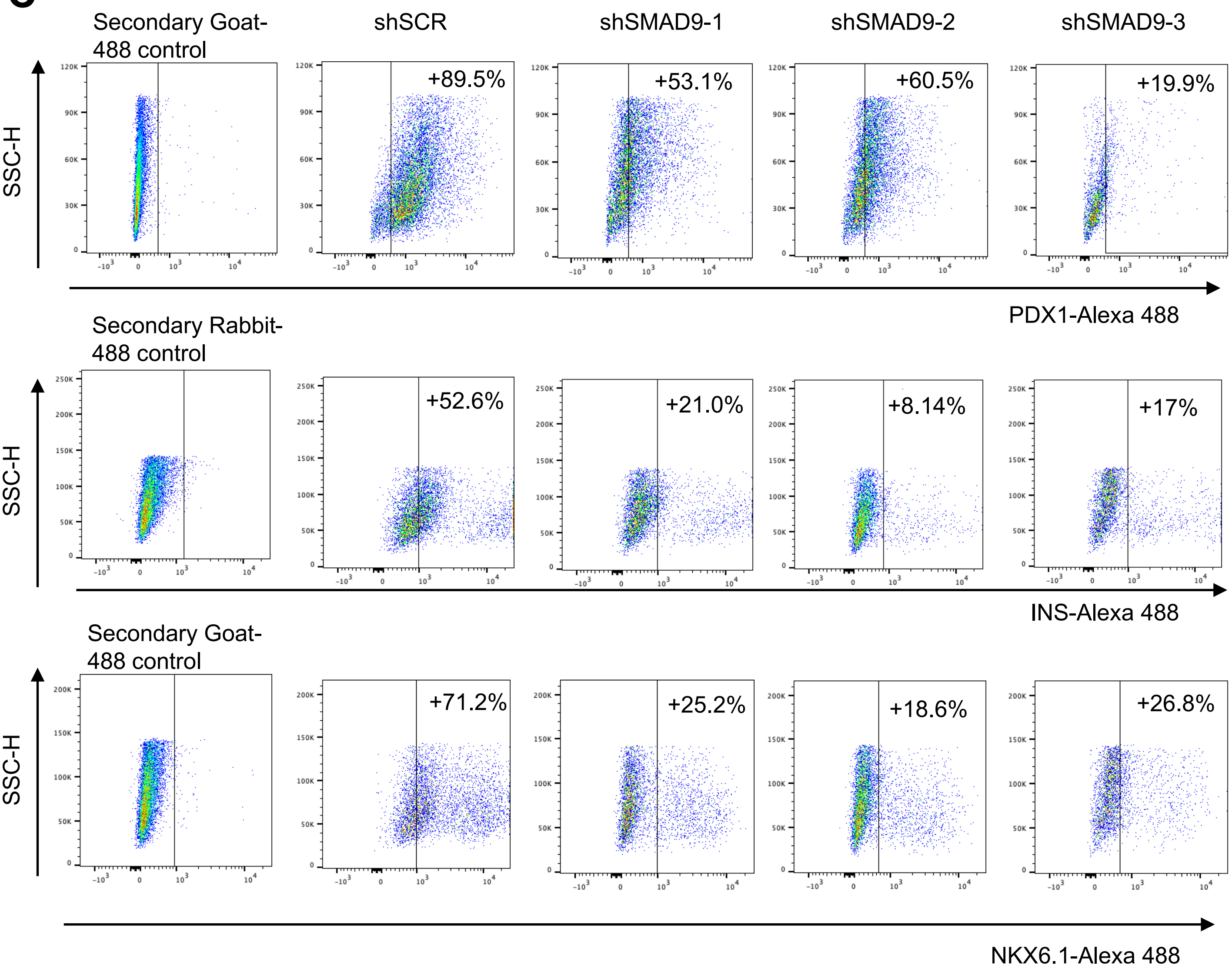

D

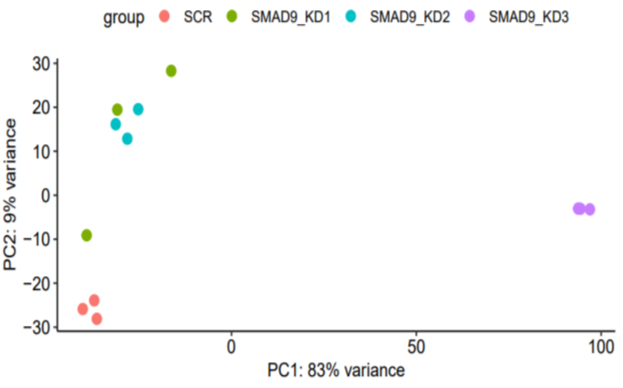

E

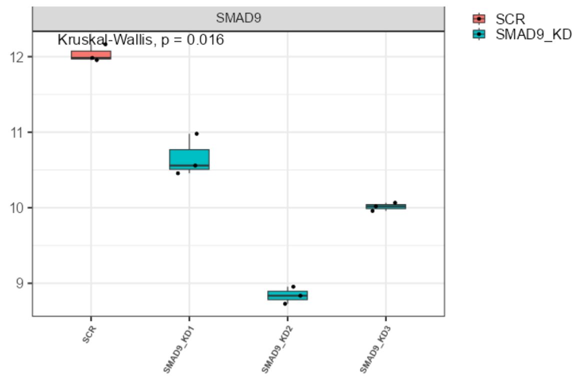

Supplementary Figure 6: Lim et al.,

F

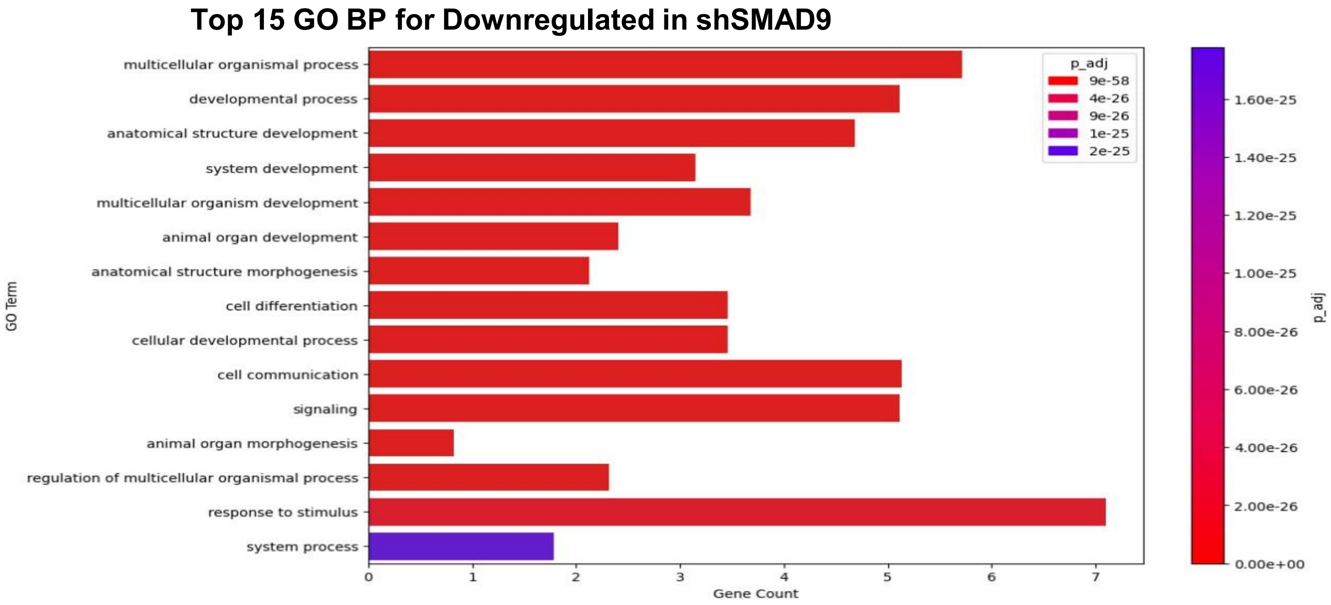

G

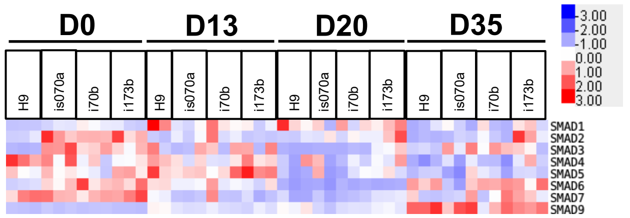

SMAD type	SMAD
BMP-specific Receptor regulated SMAD (R-SMAD)	SMAD1, SMAD5, SMAD9 (SMAD8)
Tgf-β/Activin- specific R-SMAD	SMAD2, SMAD3
Common partner SMAD (co-SMAD)	SMAD4
Inhibitory SMAD (I-SMAD)	SMAD6, SMAD7

H

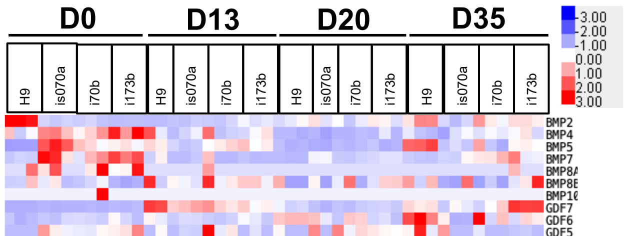

Supplementary Figure 7: Lim et al.,

A

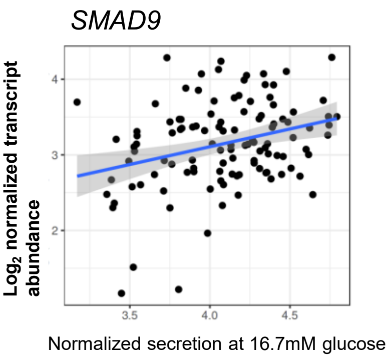

p value: 0.000088
p adj: 0.042
Coefficient: 0.594

B

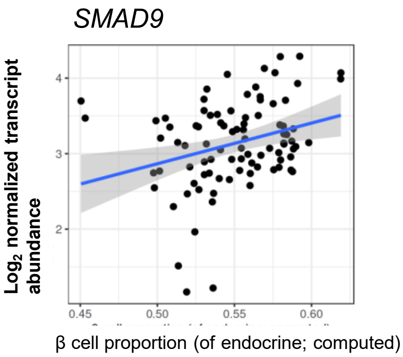

p value: 0.000514
p adj: 0.0119
Coefficient: 6.65

C

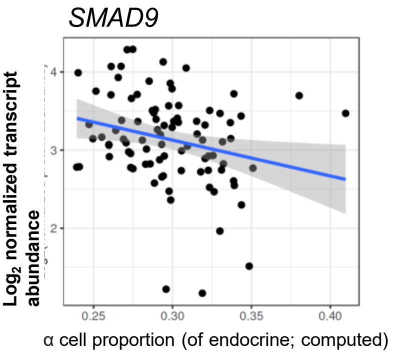

p value:0.00601
p adj:0.105
Coefficient: -5.37

D

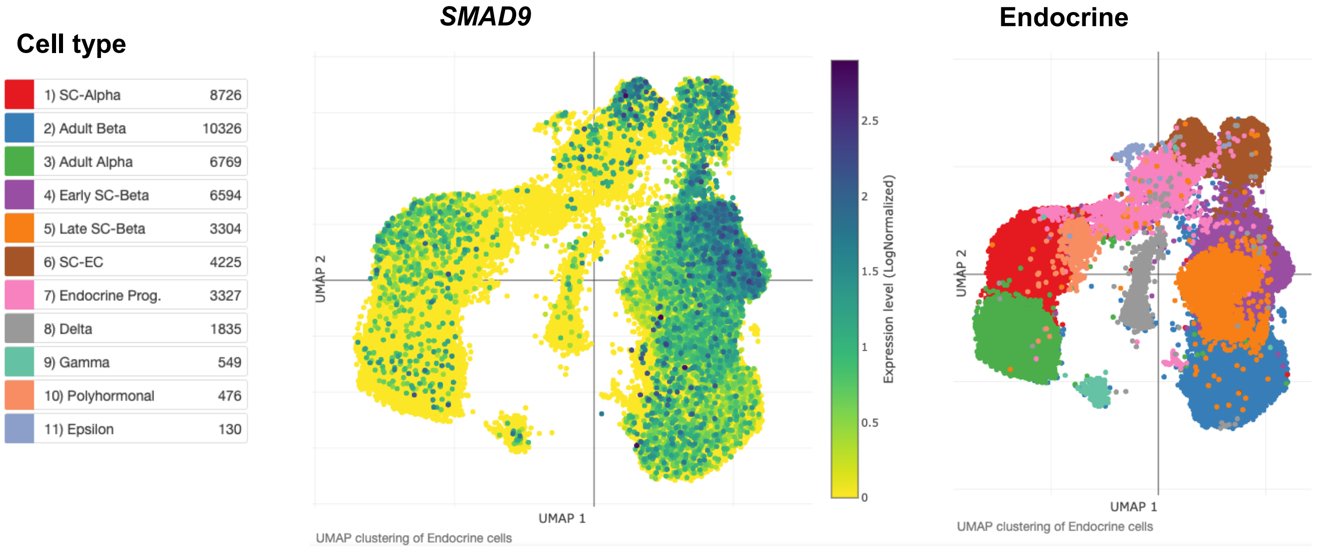

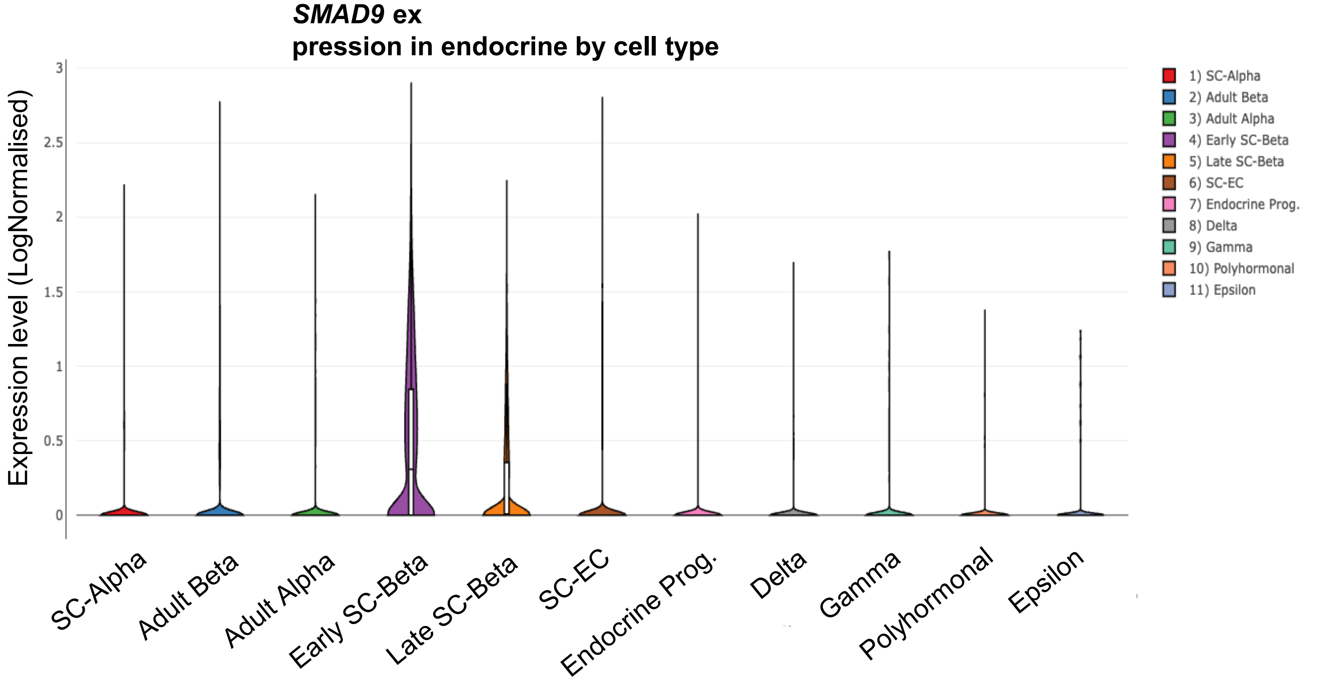

Supplementary Figure 7: Lim et al.,

E

Endocrine lineage: UMAP with slingshot trajectory

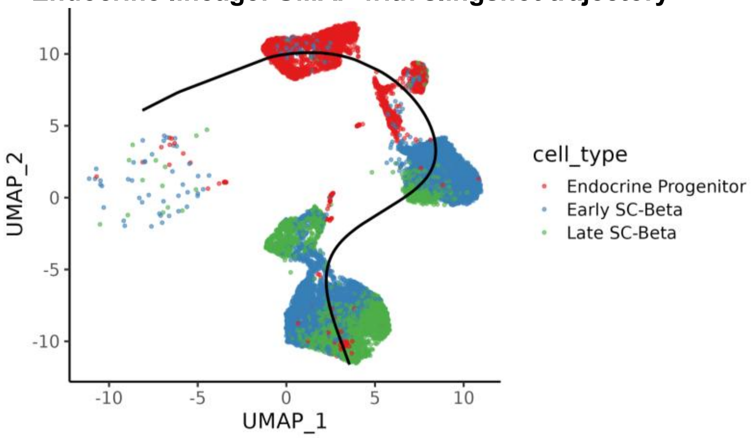

F

***SMAD9* expression along lineage pseudotime (point by cell type)**

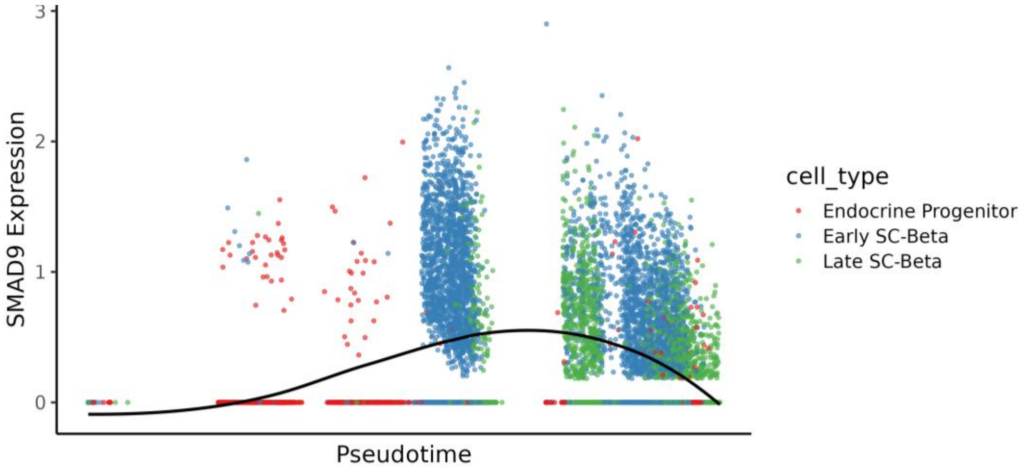

Dynamic expression of *SMAD9* (Endocrine progenitor to early SC beta)

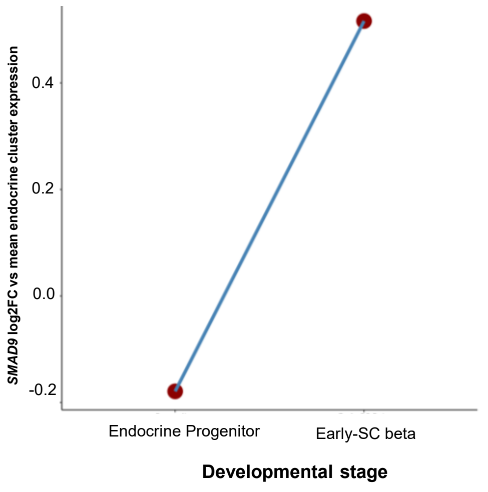

Dynamic expression of *SMAD9* (during islet maturation)

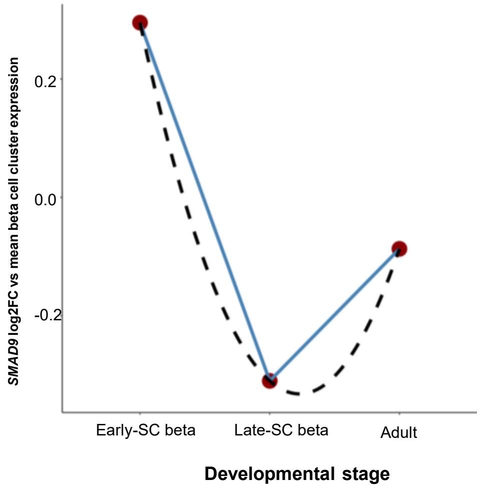

Supplement: Supplementary file 2 — Suppl Figures [file 41419_2026_8529_MOESM2_ESM.pdf]
